# Immune dysregulation in ulcerative colitis: pathogenic mechanisms and therapeutic strategies of traditional Chinese medicine

**DOI:** 10.3389/fcell.2025.1610435

**Published:** 2025-06-05

**Authors:** Xudong Tang, Yilin Huang, Ying Zhu, Yin Xu

**Affiliations:** ^1^ Department of Gastroenterology, The First Hospital of Hunan University of Chinese Medicine, Changsha, Hunan, China; ^2^ First Clinical College, Guizhou University of Traditional Chinese Medicine, Guiyang, Guizhou, China

**Keywords:** ulcerative colitis, immune dysregulation, traditional Chinese medicine, T-cell subsets, gut microbiota, gut barrier repair

## Abstract

Ulcerative colitis (UC) is a chronic inflammatory bowel disease (IBD) characterized primarily by immune dysregulation. Its pathogenesis involves multiple factors, including dysregulation of T-cell subsets, hypersecretion of pro-inflammatory cytokines, imbalance in the gut microbiota, and disruption of the intestinal barrier. Among T-cell subsets, abnormal activation of Th1 and Th17 cells, in conjunction with Treg dysfunction, significantly amplifies local pro-inflammatory signals. Pro-inflammatory cytokines, such as TNF-α, IL-6, and IL-17, exacerbate apoptosis and disrupt tight junctions (TJs) in intestinal epithelial cells (IECs), thereby creating favorable conditions for invasion by pathogenic bacteria and their metabolites. Intestinal microecological imbalance not only leads to significant alterations in the structure of the bacterial flora but also involves abnormal fluctuations in its metabolites that directly regulate intestinal immune homeostasis, a factor closely associated with the severity of inflammation and prognosis of ulcerative colitis. Recent studies have demonstrated that in the treatment of UC, traditional Chinese medicine (TCM) achieves a multi-target, multi-pathway integrated intervention by regulating immune cell differentiation, balancing inflammatory factor levels, repairing the intestinal epithelial barrier, and remodeling the structure of the bacterial flora. This article reviews the pathogenic mechanisms underlying immune dysregulation in UC and the advances in research on TCM’s role in immune regulation, anti-inflammatory repair, and flora modulation, encompassing the mechanisms of action of individual active ingredients and classic TCM compound formulas. Although some studies have preliminarily confirmed TCM’s potential to modulate immunity and repair the intestinal barrier, breakthroughs in mechanism analysis, herb standardization, and large-scale validation remain forthcoming. It is anticipated that the unique advantages of TCM will be translated into a more precise therapeutic strategy for UC through modern molecular and systems biology approaches.

## 1 Introduction

Ulcerative colitis (UC) is a chronic, progressive, and recurrent inflammatory bowel disease (IBD) characterized by persistent, nonspecific inflammation of the rectum or distal colon and superficial mucosal ulceration (Liu X. et al., 2024). The typical clinical symptoms of UC include abdominal pain, diarrhea, hematochezia, weight loss, and fatigue, and in severe cases, these symptoms can lead to complications such as intestinal perforation and colorectal cancer (Iyengar et al., 2024; Liang et al., 2024). Globally, the incidence of UC is on the rise, particularly in North America, Western Europe, and several Asian regions (e.g., Japan and Korea) (Le Berre et al., 2023). Epidemiologic data indicate that the annual incidence of UC ranges from approximately 8.8–23.1 cases per 100,000 person-years in North America and from 0.6 to 24.3 cases per 100,000 person-years in Europe (Du and Ha, 2020). In newly industrialized countries, including China, India, and regions in Latin America, both the incidence and hospitalization rates of UC are rapidly increasing (Buie et al., 2023). UC poses not only a significant threat to patients’ physical health but also imposes substantial social and economic burdens. The direct medical costs and indirect economic losses associated with UC have been estimated to amount to billions of dollars per year worldwide (Constantin et al., 2019). Despite the availability of various treatment options, including immunosuppressive agents, biologics, and intestinal surgery, the therapeutic efficacy remains limited, and the side effects of these drugs cannot be overlooked (Biedermann et al., 2024; Dai et al., 2023). For example, immunosuppressive treatments may cause adverse effects, including infections and an increased risk of tumors, whereas biologics are challenged by high treatment costs and drug resistance (Song and Wu, 2022).

**FIGURE 1:**
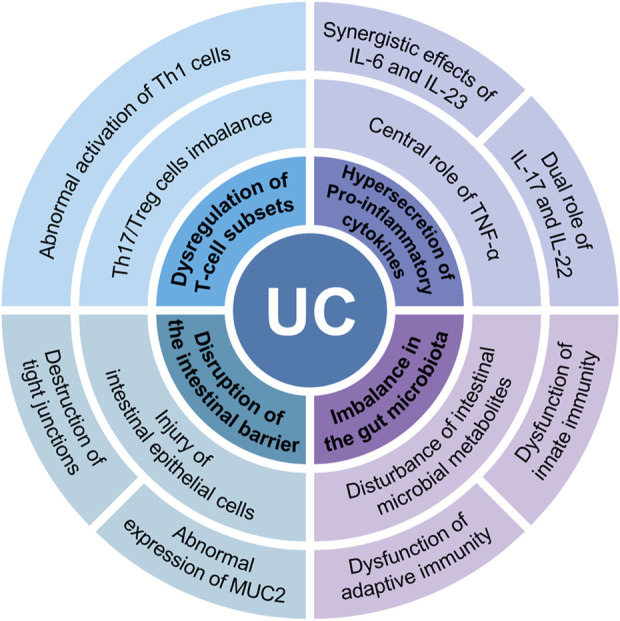
Immune dysregulation in UC is manifested in 4 main areas: dysregulation of T-cell subsets, hypersecretion of proinflammatory cytokines, imbalance in the gut microbiota, and disruption of the intestinal barrier.

The pathogenesis of UC remains incompletely understood; however, studies have demonstrated that multifactorial interactions—including genetic susceptibility, immune abnormalities, dietary factors, and environmental influences—are the primary causative factors in its development (Chen J. et al., 2024; Ananthakrishnan, 2015). In recent years, immune dysregulation has garnered significant attention as a central element in the pathogenesis of UC. As a disease primarily characterized by immune system dysfunction, the pathological progression of UC is closely associated with abnormal immune cell activation and dysregulated immune responses (Li et al., 2020; Cheng H. et al., 2024). Specifically, immune dysregulation in UC is manifested by dysregulation of T-cell subsets, hypersecretion of pro-inflammatory cytokines, imbalance in the gut microbiota, and disruption of the intestinal barrier (Figure 1). In patients with UC, the excessive differentiation of Th1 and Th17 cells and their secretion of pro-inflammatory cytokines (e.g., IFN-γ, IL-17, and TNF-α) are considered primary triggers of chronic intestinal inflammation (Cao et al., 2025; Hu et al., 2024). Furthermore, imbalances in the gut microbiota play a crucial role in immune regulation (Guo S. et al., 2021). The reduction of beneficial flora and the overgrowth of pathogenic microorganisms in the gut not only disrupt the integrity of the intestinal barrier but also exacerbate the inflammatory response by activating the immune system and promoting the recruitment of immune cells to the gut (Zhang Y-J. et al., 2015). Therefore, immune dysregulation is not only the central pathological process of UC but also a key determinant of disease progression and prognosis. An in-depth exploration of the mechanisms underlying immune dysregulation will offer valuable insights for the precise treatment of UC.

As a traditional healing system with a history of thousands of years, traditional Chinese medicine (TCM) has shown particular strengths in the treatment of UC. A large body of studies have shown that TCM exhibits a multi-component, multi-target, and multi-pathway approach in the treatment of UC. TCM can modulate overall immune function, restore intestinal immune tolerance, attenuate inflammatory responses, and exert therapeutic effects by re-establishing the balance of intestinal microecology (Li et al., 2025; Huang Y. et al., 2024). In recent years, an increasing number of clinical studies and experimental data have confirmed that TCM is efficacious in alleviating UC symptoms, prolonging remission periods, and reducing drug side effects. Notably, in regulating the immune system and enhancing intestinal microecology, certain TCM compounds and herbal formulas have shown the potential to inhibit hyperactive immune responses, repair the intestinal barrier, and restore the balance of the gut microbiota (Zhu et al., 2023; Zhou et al., 2024a). Therefore, the aim of this paper is to review the mechanisms underlying immune dysregulation in UC and to highlight the potential of active TCM ingredients and herbal formulas in its treatment.

## 2 Immune dysregulation and UC

### 2.1 Dysregulation of T-cell subsets

**FIGURE 2:**
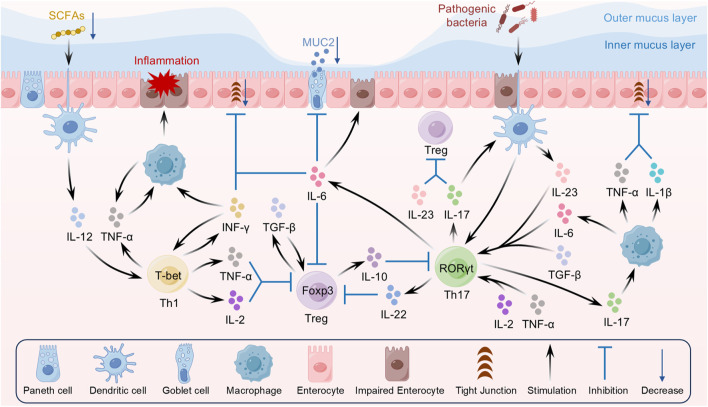
Interaction between dysregulated T cell subsets and inflammatory cytokines in UC.

The maintenance of immune homeostasis relies on a delicate balance among various immune cells, with T cell subsets playing a pivotal regulatory role. Recent studies have shown that the abnormal activation and dysfunction of T cell subsets represent a key pathogenic factor in UC (Schardey et al., 2019). Under the stimulation of inflammatory cytokines, naïve T cells differentiate into multiple lineages, including Th1, Th2, Th17, and Treg. Under normal conditions, these cells maintain mutual checks and balances through complex cytokine networks and signaling pathways, thereby preserving immune tolerance and defense. However, in UC patients, dysfunction of Th1, Th17, and Treg cells leads to abnormal local immune activation, ultimately triggering chronic inflammation in the intestinal tract (Figure 2).

#### 2.1.1 Abnormal activation of Th1 cells

Th1 cells are an important subpopulation of CD4^+^ T cells, with their differentiation driven by cytokines such as IL-12 and IFN-γ, and key functional gene expression regulated by the transcription factor T-bet (Ruan et al., 2023; Monteleone et al., 2012). IL-12 is a key cytokine regulating the differentiation of Th1 cells, as it promotes the expression of signal transducer and activator of transcription 4 (STAT4), which in turn induces the expression of the transcription factor T-bet to drive the transformation of CD4^+^ T cells into the Th1 subpopulation (Hsieh et al., 1993; Baumann et al., 2015). Under physiological conditions, Th1 cells, together with cytotoxic T lymphocytes (CTLs) and macrophages, constitute a crucial line of defense against pathogens, as their secretion of IFN-γ enhances macrophage phagocytosis and boosts antigen presentation efficiency, thereby promoting cellular immune responses (Shen et al., 2021; Thuner and Coutant, 2023). However, when Th1 cells are abnormally activated, their secretion of IFN-γ and TNF-α markedly increases, directly activating intestinal epithelial cells (IECs) and lamina propria macrophages, inducing high chemokine expression (e.g., CXCL9, CXCL10), and recruiting additional Th1 cells and neutrophils to the inflammatory site, thereby exacerbating the local inflammatory response (Elia and Guglielmi, 2018). Clinical data have shown that IFN-γ levels are elevated in the colonic mucosa of UC patients compared to healthy controls, with its expression significantly and positively correlated with disease activity (Morvaridi et al., 2025). As a key cytokine secreted by Th1 cells, IFN-γ inhibits the expression of intestinal epithelial tight junctions (TJs), resulting in impaired barrier function and increased permeability, thereby facilitating bacterial invasion and further inflammation (Boivin et al., 2009). Recent experimental results have shown that IFN-γ synergizes with TNF-α to kill IECs via the CASP8-JAK1/2-STAT1 module, thereby further disrupting intestinal barrier function (Woznicki et al., 2021). In addition to the pro-inflammatory cytokine cascade, the interaction between Th1 and Th17 cells, along with the suppression of Treg function, serves as an important driver of chronic inflammation in UC. IL-2 and TNF-α secreted by aberrantly activated Th1 cells not only inhibit Foxp3 expression in Treg cells, thereby disrupting immune tolerance, but also promote the differentiation of pathogenic Th17 cells through enhancement of the STAT4 signaling pathway (Hu et al., 2024; Xu et al., 2017). In addition, an imbalance in the gut microbiota has recently been implicated as a significant external factor contributing to Th1 cell polarization. Reduced levels of gut microbial metabolites, such as short chain fatty acids (SCFAs), in patients with UC result in decreased histone deacetylase (HDAC) inhibition, prompting dendritic cells (DCs) to secrete increased levels of IL-12 that drive the over-differentiation of Th1 cells (Shen et al., 2018). Animal model experiments have shown that probiotic intervention can effectively reduce the proportion of Th1 cells in the colon and ameliorate the symptoms of DSS-induced colitis (Guo and Lv, 2023).

#### 2.1.2 Th17/Treg cells imbalance

Th17 cells are a subset of helper T cells differentiated from CD4^+^ T cells, with their differentiation dependent on the activation of the transcription factor RORγt and synergistically regulated by cytokines such as IL-6, TGF-β, and IL-23 (Korn et al., 2009; Mickael et al., 2020). Early in the process, initial differentiation signals are provided by IL-6 and TGF-β, followed by IL-23—which enhances RORγt-mediated differentiation and pathogenicity of Th17 cells via activation of the STAT3 signaling pathway (Gomez-Bris et al., 2023). In the physiological state, Th17 cells participate in the intrinsic immune defense of the mucosal barrier through secretion of IL-17 family cytokines, playing a central role in clearing extracellular pathogens (e.g., bacteria and fungi) (Letizia et al., 2022). In addition, Th17 cells regulate neutrophil recruitment and macrophage activation, orchestrating the intensity and duration of local inflammatory responses (Fu et al., 2020). However, when Th17 cells are excessively proliferated and activated, they can induce aberrant immune responses that lead to various autoimmune diseases, including UC (Ueno et al., 2018). In the intestinal mucosa of patients with active UC, elevated numbers of Th17 cells and increased levels of associated cytokines (IL-17 and IL-23) suggest that Th17 cells play a significant role in disease activity and mucosal damage (Cao et al., 2025; Jia R. et al., 2024).

In contrast to pro-inflammatory Th17 cells, Treg cells primarily exert immunosuppressive functions, with their differentiation dependent on Foxp3 expression and activation of TGF-β and IL-2 signaling pathways (Zhong et al., 2022a; Jia et al., 2020). Under normal physiological conditions, Treg cells maintain immune tolerance by inhibiting effector T cell overactivation via cell-contact-dependent mechanisms (e.g., CTLA-4 and PD-1) and secretion of inhibitory cytokines such as IL-10 and TGF-β (Mayne and Williams, 2013). IL-10, a major inhibitory factor, effectively suppresses effector T cell proliferation and pro-inflammatory cytokine secretion, with its signaling involving activation of JAK1 and Tyk2 to mediate negative feedback regulation of the inflammatory response (Zhu et al., 2017). TGF-β, on the other hand, promotes proliferation and repair of IECs through activation of MEK1/2 signaling, thereby restoring intestinal barrier integrity and reducing immune stimulation by intestinal contents (Chen et al., 2018). In the intestinal microenvironment, Treg cells inhibit Th17 cell overproliferation and limit antigen presentation by modulating DC maturation. In addition, Treg cells adapt to the hypoxic intestinal environment through metabolic reprogramming, such as increased fatty acid oxidation, and their functional stability is closely linked to levels of gut microbiota metabolites (e.g., SCFAs) (Trapecar et al., 2020). Furthermore, a reduction or functional deficit of Treg cells leads to uncontrolled immune responses against the intestinal microbiota, thereby exacerbating chronic gut inflammation (Wang et al., 2024a).

In the pathogenesis of UC, an imbalance between Th17 and Treg cells is considered a key molecular basis. Significantly elevated levels of IL-6 and IL-23 in the intestines of UC mouse models not only promote RORγt-mediated Th17 differentiation but also inhibit TGF-β-driven Foxp3 expression, resulting in a severe Th17/Treg imbalance (Rana et al., 2021). At the transcriptional level, competitive binding of RORγt to Foxp3 within CD4^+^ T cells shifts cellular differentiation—characterized by upregulated RORγt expression in Th17 cells and Foxp3 suppression via DNA methylation modifications (e.g., SMARCA5-mediated epigenetic silencing)—thereby exacerbating immune imbalance (Wang et al., 2024b). In addition, disturbances in the gut microbiota–immunity axis play a significant role in UC pathogenesis. Pathogenic bacteria (e.g., *E. coli LF82*) activate DCs via the TLR4/MyD88 pathway, promoting Th17 cell polarization and inhibiting Treg cell differentiation; conversely, probiotics (e.g., *Lactobacillus casei R3*) help reverse the Th17/Treg imbalance by restoring SCFA levels through their metabolites (Trapecar et al., 2020; Sha et al., 2023; Huang et al., 2021). Regarding intracellular metabolic regulation, overactivation of the mTOR signaling pathway promotes Th17 cell differentiation while suppressing Treg cell immunosuppressive function; conversely, SIRT1 deacetylase enhances Treg function by modulating Foxp3 stability, although its expression is typically reduced in UC patients (Wang Y. et al., 2024; Zhu et al., 2024; Xu Y. et al., 2021). Further studies have revealed that dysregulation of the AhR-PPARγ signaling axis is critical for maintaining Th17/Treg homeostasis. AhR agonists (e.g., dimethylindole) ameliorate colitis symptoms by inhibiting Th17 differentiation and promoting Treg expansion, whereas defective PPARγ signaling disrupts the balance between RORγt and Foxp3, exacerbating the inflammatory response (Liu S. et al., 2023; Cheng et al., 2021). Meanwhile, the expression of the immune co-inhibitory molecule TIGIT is reduced in memory Th17 (mTh17) cells, weakening its negative regulatory effect on DCs and thereby exacerbating intestinal inflammation. Studies have shown that *Astragalus* polysaccharides can partially restore the balance between mTh17 and mTreg cells by upregulating TIGIT expression (Wan Q. et al., 2024).

### 2.2 Hypersecretion of pro-inflammatory cytokines

Abnormal and excessive secretion of pro-inflammatory cytokines plays a pivotal role in UC pathogenesis. Under pathological conditions, the immune system remains continuously activated, resulting in an amplified local and systemic inflammatory response in the intestine that ultimately leads to disruption of the intestinal mucosal barrier and subsequent tissue damage. Numerous studies have demonstrated that pro-inflammatory cytokines, such as TNF-α, IL-6, IL-23, IL-17, and IL-22, not only play a crucial role in localized inflammatory responses in UC but also exacerbate the immune response via systemic circulation (Figure 2).

#### 2.2.1 Central role of TNF-α

TNF-α, as a key pro-inflammatory cytokine, plays a pivotal role in regulating immune responses and inflammation. It is primarily secreted by monocyte-derived macrophages, T lymphocytes, and IECs, and regulates the immune-inflammatory response by binding to transmembrane receptors TNFR1 and TNFR2, thereby initiating a cascade of downstream signaling events (Kaur and Goggolidou, 2020; Veerasubramanian et al., 2024). Under physiological conditions, TNF-α mediates immune defense functions—including pathogen clearance and tissue repair—primarily through activation of the NF-κB and MAPK signaling pathways, while also regulating the balance between proliferation and apoptosis of IECs to maintain mucosal barrier integrity (Veerasubramanian et al., 2024; Nakase et al., 2022). In addition, TNF-α mediates the directional migration of immune cells by regulating chemokine expression, thereby functioning as a “double-edged sword” in local immune homeostasis.

However, under pathological conditions—particularly during UC development—TNF-α emerges as a significant pathological factor. First, TNF-α induces overexpression of pro-inflammatory factors such as IL-6 and IL-1β by activating the NF-κB signaling pathway, thereby establishing a positive inflammatory feedback loop that leads to persistent injury of the intestinal mucosa. Secondly, TNF-α and IL-1β contribute to the degradation of intestinal epithelial TJ proteins (e.g., occludin, claudin, ZO-1), resulting in impaired barrier function and increased mucosal permeability, which facilitates the translocation of intestinal microbiota and their metabolites, thereby activating the innate immune system and exacerbating inflammatory responses (Yu et al., 2022; Kaminsky et al., 2021). Experiments have shown that colonic TJ proteins, such as ZO-1 and Claudin-1, can be restored and colitis symptoms ameliorated by reducing TNF-α levels and inhibiting the NF-κB pathway (Song et al., 2024). In addition, TNF-α promotes the homing of α4β7 integrin-positive lymphocytes to the intestines by upregulating the expression of the vascular adhesion molecule MAdCAM-1, thereby disturbing the Th17/Treg balance and contributing to mucosal immune dysregulation (Wang et al., 2024b; Nakase et al., 2022). It was found that upregulation of colonic endothelial cell adhesion molecules can be significantly inhibited by blocking TNF-α-mediated NF-κB activation in a DSS-induced UC mouse model, thereby alleviating inflammation (Xue et al., 2023). Meanwhile, high concentrations of TNF-α can activate the caspase-8-dependent apoptotic pathway, inducing excessive apoptosis of colonic epithelial cells and downregulating the expression of the anti-apoptotic protein Bcl-2, thereby significantly reducing the repair capacity of the intestinal mucosa (Liu W. et al., 2025; Ye and Lu, 2022).

#### 2.2.2 Synergistic effects of IL-6 and IL-23

IL-6 is a pleiotropic cytokine secreted by a variety of cells, including T cells, B cells, macrophages, and endothelial cells, and its expression is markedly upregulated during inflammatory and infectious states, playing a critical role in immune regulation and tissue repair (Gong et al., 2025). Under normal physiological conditions, IL-6 regulates acute-phase inflammatory responses, promotes hematopoietic stem cell differentiation, and facilitates tissue repair primarily through activation of the JAK/STAT3 signaling pathway (Jia R. et al., 2024). In addition, IL-6 maintains immune tolerance in intestinal homeostasis by regulating the Treg/Th17 balance and enhances mucin secretion by goblet cells to protect the intestinal mucosal barrier (Leng et al., 2024; Wei et al., 2021). Unlike IL-6, IL-23, which belongs to the IL-12 family and is predominantly secreted by antigen-presenting cells (e.g., DCs and macrophages), plays a unique role in immune regulation by virtue of its heterodimeric structure composed of p19 and p40 subunits. IL-23 promotes the differentiation of Th17 cells and enhances host defense against extracellular pathogens by binding to the IL-23R/gp130 receptor complex (Aebisher et al., 2024). Meanwhile, IL-23 induces IECs to express antimicrobial peptides (AMPs) and helps maintain the dynamic balance between the gut microbiota and the host (Korta et al., 2023).

During UC pathology, IL-6 and IL-23 act synergistically via multiple signaling pathways to establish a complex, mutually reinforcing pro-inflammatory network. Specifically, IL-6 binds to its receptor IL-6R/gp130 and activates STAT3 phosphorylation, inducing junctional IECs to secrete high levels of pro-inflammatory cytokines (e.g., TNF-α, IL-1β) and chemokines (e.g., MCP-1), thereby triggering neutrophil recruitment and exacerbating local tissue inflammation (Gong et al., 2025; Zhou et al., 2020). Meanwhile, IL-6 synergizes with TGF-β to upregulate the expression of the RORγt transcription factor, prompting the differentiation of naïve T cells into Th17 cells that subsequently secrete IL-17A and IL-22, further amplifying the intestinal inflammatory response (Jia R. et al., 2024; Wei et al., 2021). In addition, IL-6 impairs the intestinal mucosal barrier and increases permeability by inhibiting MUC2 mucin synthesis and disrupting the XBP1s-mediated endoplasmic reticulum stress pathway, which results in a reduction of goblet cells and degradation of tight junction proteins (Leng et al., 2024; Zhou et al., 2020). Correspondingly, IL-23 sustains the proliferation and survival of Th17 cells by activating the STAT3 and RORγt signaling pathways, prompting them to secrete IL-17A, IL-21, and IL-22 and thereby establishing a sustained pro-inflammatory microenvironment (Wang et al., 2022; Zhong et al., 2022b). In addition, IL-23 induces Th17 cells to secrete IL-6, which further activates STAT3 and upregulates IL-23R expression, thereby establishing a positive feedback loop between IL-6 and IL-23 that exacerbates local inflammation (Wei et al., 2021; Aebisher et al., 2024). IL-23 also inhibits Treg cell differentiation by down-regulating Foxp3 expression while upregulating IL-6 and TNF-α levels, thereby disrupting the Treg/Th17 balance, leading to loss of immune tolerance and chronic inflammation (Wei et al., 2021; Zhong et al., 2022b).

#### 2.2.3 Dual role of IL-17 and IL-22

In recent years, IL-17 and IL-22 have garnered extensive attention as pivotal regulators of mucosal immune and inflammatory responses. IL-17 is a class of pro-inflammatory cytokines primarily secreted by activated Th17 cells, whereas IL-22 is produced by Th17 cells, Th22 cells, and Group 3 Innate Lymphoid Cells (ILC3), with its function tightly regulated by the local tissue microenvironment.

Under normal physiological conditions, IL-17 upholds the innate immune defense of the mucosal barrier by inducing epithelial cells to secrete AMPs (e.g., β-defensin) and chemokines (e.g., CXCL1, CXCL8) (Swedik et al., 2022; Pernomian et al., 2020). IL-17 facilitates neutrophil recruitment via activation of the STAT3 signaling pathway and augments host pathogen clearance (Lv et al., 2023). In addition, it attenuates intestinal inflammation by upregulating atypical M2 macrophage subpopulations (Nishikawa et al., 2014). IL-22, on the other hand, activates the STAT3 and MAPK pathways by binding to its receptor, thereby promoting IECs proliferation, mucus secretion, and the expression of TJ proteins to safeguard intestinal barrier integrity (Huang et al., 2022). IL-22 also induces the expression of Reg3β and Reg3γ, thereby accelerating mucosal repair following injury. In models of chronic inflammation or infection, neutralization of IL-22 has been shown to compromise intestinal barrier integrity and exacerbate tissue damage (Lo et al., 2019). Notably, a functional synergism exists between IL-17 and IL-22 under homeostatic conditions; for example, IL-22 can further potentiate the barrier immune response by enhancing IL-17-mediated AMPs expression (Swedik et al., 2022).

However, in UC, the aberrant expression and dysregulated signaling of IL-17 and IL-22 convert their roles from protective to pro-inflammatory and tissue-damaging. IL-17 stimulates the release of IL-6, TNF-α, and IL-1β from IECs and lamina propria macrophages via activation of the NF-κB and MAPK pathways, thereby creating a chronic inflammatory microenvironment (Yu et al., 2023; Yin et al., 2021). Clinical studies have shown that IL-17 expression is markedly elevated in the serum and inflamed bowel mucosa of active UC patients and positively correlates with disease activity (Fujino et al., 2003). Second, IL-23 continuously promotes IL-17 secretion by upregulating RORγt expression in Th17 cells, while IL-17 in turn augments IL-23 production by antigen-presenting cells (e.g., DCs), thereby establishing a self-amplifying inflammatory loop. IL-23 inhibitors have been shown to significantly reduce mucosal IL-17 levels in UC patients by interrupting this loop (Gottlieb and Sands, 2022). In addition, IL-22 induces IL-17C overexpression in epithelial cells via activation of the AP-1 transcription factor and p38 MAPK signaling, which further stimulates Th17 cells to secrete IL-17A, thereby creating an inflammatory amplification loop between the epithelium and immune cells (Swedik et al., 2022). Abnormal elevation of IL-22 also leads to excessive IECs proliferation and goblet cells depletion, resulting in mucus layer thinning and increased barrier permeability (Huang et al., 2022). In addition, IL-17 and IL-22 together disrupt immune homeostasis and exacerbate the Th17/Treg imbalance by inhibiting Treg cell differentiation (via Foxp3 downregulation) and suppressing Th1 cell function (by reducing IFN-γ secretion), respectively (Lv et al., 2023). It is worth mentioning that under co-stimulation by TNF-α and IL-17A, IECs secrete IL-17C, which activates STAT3 signaling in Th17 cells via the IL-17RE receptor, thereby further driving the release of IL-17A and IL-22 and forming a vicious cycle (Swedik et al., 2022). Meanwhile, during the active phase of UC, the pro-repair isoform of IL-22 (e.g., IL-22/STAT3/Reg3γ axis) shifts to a pro-inflammatory isoform (e.g., IL-22/AP-1/IL-17C axis), a functional polarization closely linked to intestinal microbiota dysbiosis and Dectin-1 signaling activation (Azizollah et al., 2024).

### 2.3 Imbalance in the gut microbiota

**FIGURE 3:**
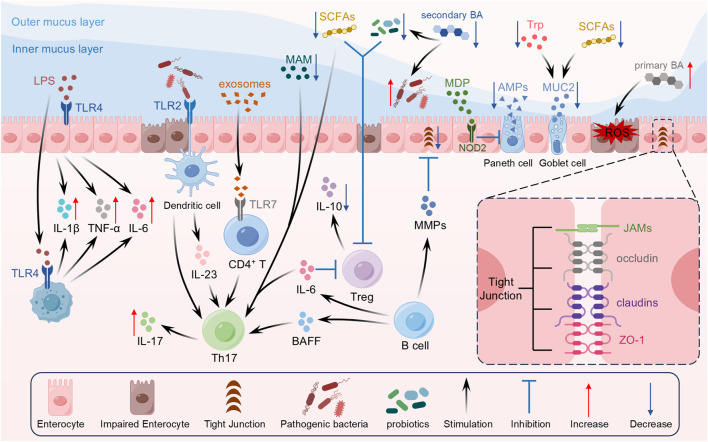
Gut microbiota imbalance and intestinal barrier damage caused immune dysregulation in UC.

As the largest microecosystem in the human body, the intestinal microbiota plays an indispensable role in maintaining host physiological homeostasis and regulating immune function (Ma et al., 2019). This community predominantly comprises *Bacteroidetes*, *Firmicutes*, *Proteobacteria*, *Actinobacteria*, and *Verrucomicrobia*, with *Bacteroidetes* and *Firmicutes* constituting over 90% of the total flora and primarily colonizing the colon and rectum (Schroeder and Bäckhed, 2016; Rajilić-Stojanović et al., 2007). In recent years, a large body of experimental evidence has demonstrated that the intestinal microbiota is not only involved in energy metabolism but also serves as a crucial modulator of innate and adaptive immune responses, thereby contributing to the maintenance of intestinal barrier integrity and overall host health (Ansaldo et al., 2021; Wang et al., 2024d). In addition, metabolites derived from the microbiota—such as short‐chain fatty acids (SCFAs), bile acids (BAs), and tryptophan (Trp)—are vital for maintaining the gut barrier and regulating immune homeostasis (Franzosa et al., 2019; Lloyd-Price et al., 2019) (Figure 3).

#### 2.3.1 Imbalance of gut microbiota and dysfunction of innate immunity

The intestinal innate immune response primarily depends on IECs and resident immune cells (e.g., macrophages, DCs, and neutrophils) to mount rapid responses to external stimuli. In the pathogenesis of UC, an imbalance in the gut microbiota is closely associated with aberrant activation of pattern recognition receptors (PRRs). As two core families of PRRs, Toll-like receptors (TLRs) and nucleotide-binding oligomerization domain-like receptors (NLRs) are activated by recognizing microbe-associated molecular patterns (MAMPs) and play pivotal roles in regulating innate immunity.

TLR4 is particularly important in UC pathology as a key receptor for recognizing Gram-negative bacterial lipopolysaccharides (LPS). When the intestinal microbiota is dysbiotic, Gram-negative bacteria such as *Proteobacteria* proliferate excessively and release copious amounts of LPS, which binds to TLR4 on IECs and lamina propria macrophages, thereby activating the MyD88-dependent NF-κB signaling pathway and inducing the expression of pro-inflammatory cytokines (e.g., TNF-α, IL-6, and IL-1β) and chemokines (e.g., CXCL1 and CXCL8) (Chen et al., 2020; Mirpuri et al., 2014; Miao et al., 2021). Clinical observations have shown that TLR4 expression is markedly upregulated in the colonic mucosa of UC patients and positively correlates with disease activity (Xu N. et al., 2021). TLR2 mainly recognizes lipoproteins from Gram-positive bacteria and exerts a bidirectional regulatory role in maintaining intestinal homeostasis. When the microbiota is dysbiotic, the abnormal colonization of pathogenic bacteria (e.g., *Klebsiella pneumoniae*) and alterations in their lipoprotein structures may lead to overactivation of TLR2, prompting dendritic cells to secrete abundant IL-23 that induces Th17 cell differentiation and IL-17A release, thereby compromising epithelial barrier function (Atarashi et al., 2017). Conversely, probiotics (e.g., *Lactobacillus*) can enhance the suppressive function of Treg cells via a TLR2-dependent mechanism (Al-Sadi et al., 2021).

NOD2 is a critical cytoplasmic receptor for recognizing bacterial muramyl dipeptide (MDP), and its loss of function or mutation is recognized as a significant genetic risk factor for UC. NOD2 deficiency not only reduces the capacity of Paneth cells to secrete AMPs such as α-defensins—thereby facilitating commensal bacterial penetration and subsequent TLR4 activation—but is also closely associated with a diminished response to probiotic therapy in UC patients, suggesting a complex interplay between genetic factors and bacterial dysbiosis (Wehkamp et al., 2008; Liu et al., 2022). Meanwhile, NOD2 clears intracellular invading pathogens (e.g., adherent-invasive *Escherichia coli* [AIEC]) by regulating the autophagy-related gene ATG16L1, and its functional defects can lead to abnormal AIEC proliferation and subsequent activation of NLRP3 inflammasomes (Zheng et al., 2022; Tran et al., 2023). Imbalances in the gut microbiota also generate various metabolites—such as reactive oxygen species (ROS), hydrogen sulfide (H_2_S), and pathogen-associated molecules (e.g., bacterial flagellin epitopes)—which activate the NLRP3 inflammasome via a two-step signaling mechanism: the first signal, induced by the TLR4/NF-κB pathway, upregulates NLRP3 and pro-IL-1β expression, while the second signal—mediated by potassium efflux or mitochondrial ROS—promotes inflammasome assembly and caspase-1 activation, leading to the maturation of IL-1β and IL-18 (Zhou et al., 2011; He et al., 2016; Swanson et al., 2019). In colon biopsies of UC patients, levels of both NLRP3 and IL-1β are markedly elevated; moreover, application of the NLRP3 inhibitor MCC950 in animal models significantly alleviates colonic inflammatory damage (Liu et al., 2017; Perera et al., 2018).

#### 2.3.2 Imbalance of gut microbiota and dysfunction of adaptive immunity

Intestinal adaptive immunity is a specific response mediated by T and B lymphocytes that recognize gut microbiota and their metabolites via antigen-presenting cells, thereby establishing a protective mechanism characterized by antigen specificity and immune memory crucial for maintaining host homeostasis (Owen and Mohamadzadeh, 2013). Gut microbiota imbalance triggers mucosal immune abnormalities and inflammatory injury by disrupting the activation, differentiation, and function of T and B cells.

Dysregulation of T cell subsets constitutes a central feature of UC immunopathology. Specifically, the symbiotic bacterium *Faecalibacterium prausnitzii*, whose abundance is markedly reduced in UC patients, secretes a microbial anti-inflammatory molecule (MAM) that curtails Th17 cell differentiation by inhibiting the IL-6/STAT3 signaling pathway (Zhao et al., 2021; Ratajczak et al., 2019; Qiu et al., 2013). In contrast, pathogenic bacteria (e.g., AIEC) stabilize and expand the Th17 cell population by activating dendritic cells and promoting IL-23 secretion, which in turn triggers robust IL-17A production and exacerbates mucosal inflammation (Leccese et al., 2024). In addition, the gut microbiota further promotes Th17 cell polarization by secreting exosomes that carry 16S rRNA fragments capable of directly activating lamina propria CD4^+^ T cells via TLR7 (Zhou et al., 2021; Harrell et al., 2019). Meanwhile, the recent discovery of IL-17-producing Treg cells suggests a delicate dynamic balance between Th17 and Treg cell populations. Metabolites derived from *Clostridiales* enhance Treg cell IL-10 secretion, thereby suppressing inflammatory responses and helping to maintain immune homeostasis (Cui et al., 2024). However, *clostridial* abundance is generally reduced in UC patients compared with healthy individuals, thereby weakening Treg cell regulatory functions and predisposing to uncontrolled inflammatory cytokine release (Rossen et al., 2015).

B cells also play a crucial role in maintaining gut microbiota homeostasis. In healthy individuals, intestinal IgA^+^ B cells maintain microbial balance by secreting polyclonal IgA to neutralize pathogens, whereas in UC patients, aberrant IgA class switching leads to increased secretion of pathogenic IgG antibodies (e.g., anticommensal bacterial antibodies), which disrupt the intestinal epithelial barrier and facilitate translocation of bacterial antigens to the lamina propria, thereby activating T cells and triggering an autoimmune response (Fleming et al., 2022; Sengupta and Sen, 2024). In addition, IL-6 and B-cell activating factor (BAFF) secreted by B cells promote Th17 cell differentiation while concurrently suppressing Treg cell function, thereby exacerbating mucosal inflammation (Kim, 2021). Some B cells also directly disrupt tight junction proteins in the intestinal epithelium by secreting matrix metalloproteinases (MMPs) and other pro-inflammatory factors, thereby increasing mucosal permeability and creating a vicious inflammatory cycle (Kim, 2021; Inciuraite et al., 2024). Notably, the marked reduction in phage abundance in UC patients impairs the lytic capacity against pathogenic bacteria (e.g., *E. coli*), indirectly promoting aberrant B-cell-mediated autoantibody production (Bai et al., 2022; Özdirik et al., 2021).

#### 2.3.3 Disturbance of intestinal microbial metabolites

The gut microbiota not only regulates immune responses through direct cell-to-cell interactions, but its metabolites also play multifaceted roles in UC development. Under normal conditions, gut microbial metabolites—such as SCFAs, BAs, and Trp—help maintain luminal pH balance, stabilize the microecological barrier, and modulate inflammatory responses by providing energy to epithelial cells, regulating TJ proteins, and supporting goblet cells differentiation, respectively. However, in UC, deficiencies or metabolic disorders of these metabolites can lead to impaired intestinal barrier function, aberrant release of pro-inflammatory factors, and overgrowth of pathogenic bacteria, collectively driving disease onset and progression.

Dietary fiber and other indigestible carbohydrates are fermented by intestinal commensal bacteria to produce SCFAs (Blaak et al., 2020). SCFAs include acetate, propionate, and butyrate; acetate and propionate are predominantly produced by *Bacteroidetes*, whereas butyrate is largely derived from the metabolic activities of *Firmicutes* (Vogt et al., 2015). Butyrate, a major energy source for colonocytes, is crucial for maintaining the repair and barrier integrity of IECs (Hodgkinson et al., 2023). High concentrations of butyrate not only inhibit the adhesion and colonization of pathogenic bacteria, thereby reducing infection risk, but also exert potent anti-inflammatory effects by preserving Th17/Treg cell homeostasis through HDAC inhibition, promotion of Foxp3 expression, and blockade of the IL-6/STAT3/IL-17 signaling pathway (Akhtar et al., 2022; Zhou et al., 2018). However, in UC patients, butyrate levels are markedly reduced, resulting in insufficient HDAC inhibition, decreased MUC2 mucin secretion and tight junction protein expression, and consequently increased intestinal mucosal permeability (Langhorst et al., 2020; Yin et al., 2024). Notably, butyrate, a ligand for SCFAs-sensing G-protein-coupled receptors (GPCRs), exerts anti-inflammatory signaling by binding to GPR41, GPR43, and GPR109A (Siddiqui et al., 2024; Kalkan et al., 2025). Studies have demonstrated that butyrate activates the GPR109A signaling pathway to enhance the anti-inflammatory functions of colonic macrophages and dendritic cells and to promote the differentiation of Treg cells (Xiao et al., 2022; Song et al., 2025). Furthermore, butyrate alleviates intestinal inflammation by activating GPR43, which upregulates granzyme B expression in CD4^+^ T cells, particularly IL-10-producing Th1 cells (Yang W. et al., 2024). Clinical studies have shown that the abundance of butyrate-producing bacteria (e.g., *Clostridium globosum* and *Clostridium fascicularis*) in the feces of UC patients is reduced, with lower butyrate concentrations correlating negatively with disease activity and inflammation severity (Kumari et al., 2013). In addition, SCFAs deficiency impairs Treg cell differentiation and fails to inhibit Th17 cell polarization, leading to an abnormal increase in pro-inflammatory factors (e.g., IL-17 and IFN-γ) that exacerbate intestinal inflammation (Bajaj et al., 2024; Kovtonyuk and McCoy, 2023).

BAs are amphiphilic molecules synthesized by the liver from cholesterol and play a key role in regulating glucolipid metabolism and immune responses (Wang et al., 2021). Upon entering the intestine, primary BAs are metabolized by the intestinal flora—first undergoing deconjugation by bile salt hydrolase (BSH) and subsequently 7α-dehydroxylation—to generate secondary BAs (Cai et al., 2022). Under physiological conditions, secondary BAs induce the expression of antimicrobial factors (e.g., nitric oxide synthase and IL-18), thereby maintaining the intestinal microecological barrier (Inagaki et al., 2006). Secondary BAs also regulate intestinal metabolism and immune responses through activation of signaling pathways such as the G protein-coupled bile acid receptor 1 (TGR5) and farnesoid X receptor (FXR), and inhibit pathogenic bacterial proliferation to maintain microbiota homeostasis (Hang et al., 2019). In UC patients, reduced secondary BA levels lead to insufficient FXR activation, thereby impairing its anti-inflammatory effects through NF-κB pathway inhibition (Sun C. et al., 2024; Liu Y. et al., 2025). Conversely, excessive accumulation of primary BAs induces ROS generation and caspase-3 activation in mitochondria, thereby promoting apoptosis of colonic epithelial cells (Liu Y. et al., 2025; Chen and Ho, 2023). In addition, an altered BAs composition disrupts the microbiota-host balance by promoting the overgrowth of pathogenic bacteria (e.g., *Proteobacteria*) while inhibiting butyrate-producing bacteria (e.g., *Faecalibacterium*), thereby creating a pro-inflammatory microenvironment (Yin et al., 2024; Sun C. et al., 2024).

Trp, an essential amino acid, is metabolized by gut microbiota into various bioactive compounds—such as indole and its derivatives (e.g., indole propionic acid, indole acetic acid), 5-hydroxytryptamine (5-HT), and kynurenine—following dietary intake (Agus et al., 2018). Normally, Trp metabolites regulate the intestinal immune response and maintain barrier integrity by activating the aryl hydrocarbon receptor (AhR), which promotes goblet cell differentiation and mucus secretion. However, UC patients often exhibit a marked reduction in indole metabolites, resulting in impaired AhR signaling, decreased goblet cell numbers, and mucus layer thinning, thereby increasing the risk of pathogen colonization (Deng et al., 2024; Wang et al., 2024e). A significant decrease in indole and indole propionic acid levels has also been observed in a DSS-induced colitis mouse model (Cheng W. et al., 2024). Meanwhile, enhanced indoleamine 2,3-dioxygenase 1 (IDO1) activity converts Trp to kynurenine, promotes Th17 cell differentiation and IL-22 secretion, and drives chronic inflammation (Kovtonyuk and McCoy, 2023; Alexeev et al., 2018). Clinically, a negative correlation between serum Trp levels and disease activity in IBD patients has been observed, suggesting that Trp deficiency is closely linked to inflammation onset and progression (Nikolaus et al., 2017).

### 2.4 Disruption of the intestinal barrier

Intestinal barrier primarily composed of synergistic physical and chemical components: the physical barrier—including IECs and their TJs—and the chemical barrier, represented by extracellular mucus (Zhou et al., 2024a; Zong et al., 2023). An intact intestinal barrier blocks exogenous pathogens while limiting the penetration of endogenous toxins and bacterial products. When barrier function is impaired, bacteria, toxins, and other harmful substances readily penetrate the epithelium into the submucosa, triggering abnormal local immune activation and a chronic inflammatory response that plays a central role in ulcer pathogenesis (Turner, 2009). (Figure 3).

#### 2.4.1 Injury of intestinal epithelial cells

IECs, the core functional units of the physical barrier, comprise various cell types—including enterocytes, goblet cells, Paneth cells, enteroendocrine cells (EECs), and LGR5^+^ intestinal stem cells (ISCs)—which are organized in an orderly and synergistic manner (Zheng and Duan, 2023). Their apical surface forms a continuous physical barrier via TJ proteins, while the basolateral surface establishes a dynamic interface with immune cells through adhesion molecules, collectively maintaining intestinal homeostasis (Li H. et al., 2023). Under normal conditions, enterocytes are primarily responsible for nutrient uptake and metabolism (Pan X. et al., 2024); goblet cells secrete the mucin MUC2 to form a uniform mucus layer that blocks direct pathogen contact and provides an ecological niche for commensals (Gustafsson and Hansson, 2025); Paneth cells regulate gut microbiota balance and enhance antimicrobial defense by secreting AMPs such as α-defensins (Wang et al., 2018); and LGR5^+^ ISCs continuously replenish and repair the epithelium via the Wnt/β-catenin signaling pathway (Ayansola et al., 2024). Moreover, IECs secrete cytokines and chemokines that alert the mucosal immune system to local damage and coordinate inflammatory as well as repair processes (Kuhn et al., 2014).

In pathological states, injury to IECs triggers UC onset and progression via multiple mechanisms. First, oxidative stress plays a critical role. Activation of the myeloperoxidase (MPO) system leads to excessive ROS production, which directly damages mitochondrial DNA in IECs and induces endoplasmic reticulum stress (ERS) and apoptosis via the mitochondrial pathway (Wan et al., 2022). Single-cell sequencing has revealed that oxidative stress-related genes (e.g., GPX4 and SOD2) are significantly downregulated in IECs from UC patients (Hsu et al., 2023). Second, an imbalance in cell death patterns in IECs is another key mechanism in UC development. Recent studies have found that the P2Y14 receptor (P2Y14R) activates RIPK1 via the cAMP/PKA/CREB axis, inducing necroptosis in IECs and triggering the release of damage-associated molecular patterns (DAMPs) that further activate macrophages and neutrophils, exacerbating inflammation (Liu C. et al., 2024; Wang et al., 2024f). Regenerative dysfunction of ISCs is also critical; the inflammatory microenvironment reduces LGR5^+^ ISC numbers by inhibiting Wnt/β-catenin signaling and impairs intestinal regeneration by skewing differentiation toward goblet cells via Notch pathway dysregulation (Ou et al., 2024). Cytokine imbalance further contributes to IECs damage: IL-1β secretion by myeloid cells inhibits neuregulin 1 (NRG1) expression in fibroblasts, thereby blocking EGFR-mediated repair signaling (Qiu et al., 2024), whereas IL-22 promotes MUC2 synthesis via STAT3 activation, although its protective effect is modulated by autophagy levels (Nong et al., 2024).

#### 2.4.2 Destruction of tight junctions

TJs of IECs are an essential multiprotein complex located on the apical surface, comprising transmembrane proteins (e.g., occludin, claudin family proteins, and junctional adhesion molecules [JAMs]) and cytoplasmic scaffolding proteins (e.g., ZO-1, ZO-2, ZO-3), which together form a dynamic structure functioning as both a “molecular fence” and a “molecular sieve” (Kaminsky et al., 2021; Suzuki, 2020). Among these, occludin—the first identified TJ transmembrane protein—is primarily located at cell–cell contact sites, where it “seals” intercellular gaps and regulates the permeability of ions and small molecules (Furuse et al., 1993). The claudin family comprises 27 isoforms with specific roles in intercellular junctions; for example, claudin-1 enhances intercellular sealing and modulates Notch signaling to regulate epithelial homeostasis (Chen et al., 2024b; Pope et al., 2014), whereas claudin-2 functions as a cation-selective channel, and its overexpression is positively correlated with increased permeability and inflammatory activity in UC (Van Itallie and Anderson, 2006; Raju et al., 2020). In addition, JAMs, particularly JAM-A, play a key role in regulating inter-epithelial permeability; their high expression under normal conditions helps maintain barrier function, whereas their levels are significantly decreased in UC patients and DSS-induced colitis models (Vetrano et al., 2008). As a scaffolding protein with multiple domains, ZO-1 interacts with transmembrane proteins and the cytoskeleton via its PDZ domain, providing a platform for TJ assembly and stabilization. It not only participates in TJ reconstruction but also regulates cell proliferation and polarity, thereby promoting mucosal repair (Kuo et al., 2021; Yoseph et al., 2016).

In UC patients, the TJ structure is disrupted by multiple factors, constituting an important mechanism for disease exacerbation. First, pro-inflammatory cytokines mediate abnormal TJ protein expression. TNF-α, IL-6, and IFN-γ significantly downregulate TJ proteins (e.g., occludin and ZO-1) via activation of STAT3/NF-κB signaling, while concurrently upregulating pore-forming proteins such as claudin-2, thereby increasing barrier permeability abnormally (Rawat et al., 2020; Li et al., 2021). In addition, IL-1β inhibits claudin-1 expression by modulating microRNAs (e.g., miR-195-5p), further disrupting barrier integrity (Scalavino et al., 2022). Second, dysregulation of the interactions between the gut microbiota and TJs also plays a key role in UC pathogenesis. UC patients often exhibit mucus layer defects (e.g., reduced MUC2 expression), allowing commensal bacteria direct access to IECs that activate the TLR4/MyD88 pathway and suppress occludin transcription (Zhang et al., 2024a; Jin et al., 2024). Meanwhile, serine proteases secreted by pathogenic bacteria (e.g., *Clostridium nucleatum*) degrade ZO-1, whereas probiotics enhance claudin-5 expression and facilitate barrier repair by upregulating IL-34 (Jin et al., 2024; Chen et al., 2025). Furthermore, gut microbiota metabolites such as SCFAs regulate claudin-4 and occludin expression via activation of the AhR pathway; however, AhR ligand levels (e.g., Trp metabolites) are markedly decreased in UC (Wang X. et al., 2024; Li Y-Y. et al., 2022). In addition, aberrant mTOR pathway activation inhibits autophagy-related proteins (e.g., LC3-II), impairing clearance of damaged TJ proteins and further exacerbating barrier dysfunction. Rapamycin can restore autophagy and promote TJ protein regeneration by inhibiting mTOR, a mechanism involving complex regulation via the AMPK/mTOR/ULK1 pathway (Xu et al., 2024; Pan S-M. et al., 2024). Oxidative stress is also a critical factor in TJ disruption; excess ROS and reactive nitrogen species (RNS) can modify proteins such as occludin via nitration or chlorination, destabilizing their conformation and causing ZO-1 to dissociate from the cytoskeleton (Zhang Q. et al., 2025; Cartwright et al., 2024). At sites of UC inflammation, abundant MPO catalyzes chlorination modifications of proteins (e.g., claudin-3), inducing TJ mislocalization (Cartwright et al., 2024). Once the TJ structure is compromised, intestinal luminal antigens (e.g., LPS) can traverse the paracellular pathway into the lamina propria, activate dendritic cells, promote Th17 differentiation, and trigger IL-17 secretion that further inhibits claudin-1 expression (Rawat et al., 2020; Mansouri et al., 2025).

#### 2.4.3 Abnormal expression of MUC2

MUC2 is a high–molecular-weight mucin specifically secreted by intestinal goblet cells and constitutes a major structural component of the colonic mucus layer. Its core function depends on a highly glycosylated structure that forms a gel-like matrix to maintain the integrity of the intestinal chemical barrier. Under normal conditions, the double-layered mucus—comprising an outer loose layer and an inner dense layer—formed by MUC2 secretion not only prevents direct contact between pathogens and IECs but also enhances innate immune defense by trapping secretory IgA and AMPs (Yao D. et al., 2021; Wei et al., 2023). Moreover, its complex glycosylation provides abundant metabolic substrates for commensal flora, thereby maintaining intestinal microbial balance, and it participates in regulating goblet cell autophagy to promote epithelial repair and preserve dynamic barrier function (Zhang et al., 2024a).

Abnormalities in MUC2 expression and function have emerged as a key molecular mechanism in UC development. Defective O-glycosylation of MUC2 reduces mucus viscoelasticity, facilitating pathogenic bacteria (e.g., *E. coli*) penetration and direct invasion of IECs, while inducing NF-κB signaling that contributes to the release of pro-inflammatory factors (e.g., TNF-α, IL-6) (He et al., 2023). Meanwhile, structural defects in glycan chains weaken interactions with commensal bacteria and contribute to a shift toward a pro-inflammatory microbial phenotype; clinical data indicate that decreased MUC2 O-glycosylation in early UC is negatively correlated with virulence gene expression (Wei et al., 2023; Wang T. et al., 2024). Furthermore, MUC2 missense mutations (e.g., as observed in the Winnie mouse model) trigger protein misfolding, leading to persistent ERS and activation of the unfolded protein response (Wilson et al., 2020; Talà et al., 2022). ERS induces goblet cell apoptosis via the PERK-eIF2α pathway and inhibits normal MUC2 secretion, resulting in mucus layer disruption and the release of allergens (e.g., IL-33) that drive a Th2-type inflammatory response (Wilson et al., 2020; Singh et al., 2020). Microbiota dysbiosis (e.g., enrichment of *Proteobacteria*) further accelerates MUC2 degradation, establishing a vicious cycle of barrier disruption, bacterial translocation, and inflammation amplification (Wei et al., 2023; Cheng et al., 2022). Abnormal MUC2 accumulation inhibits autophagic flux in goblet cells, leading to lysosomal dysfunction and ROS buildup; autophagy defects then inhibit MUC2 transcription via mTORC1 feedback and reduce AMPK phosphorylation, impairing cellular energy stress responses (Zhang et al., 2024a; Cai et al., 2021). Notably, interactions among receptor signaling pathways also regulate MUC2 secretion. Activation of the adenosine A3 receptor (ADORA3) stimulates MUC2 secretion via the cAMP/PKA pathway; however, its expression is significantly downregulated in UC patients, resulting in insufficient mucus production. Conversely, nicotinic receptor agonists enhance MUC2 secretion by inhibiting ERK phosphorylation, suggesting a potential role for cholinergic neuromodulation in mucus barrier repair (Singh et al., 2020; Zeng et al., 2024).

## 3 Treatment of UC with TCM

### 3.1 TCM active ingredients

#### 3.1.1 Polysaccharides

*Polygonatum cyrtonema* polysaccharides (PCPs), primarily derived from the dried rhizomes of the *Liliaceae* plant *P. cyrtonema*, exhibit various biological activities, including immunomodulatory, antioxidant, anti-inflammatory, and intestinal microbiota–modulating effects (Yuan et al., 2025; Shan et al., 2024). PCPs have been found to alleviate DSS-induced colitis by inhibiting pro-inflammatory factor expression, enhancing antioxidant enzyme activity, and restoring the integrity of TJ proteins in the intestinal mucosal barrier. Moreover, macrogenomic and metabolomic analyses have confirmed that PCPs enrich beneficial bacteria (e.g., *Akkermansia muciniphila* and *Muribaculum*), inhibit pathogenic bacteria (e.g., CAG-873), and significantly reverse DSS-induced microbiota dysbiosis. The efficacy of PCPs depends on the integrity of the gut microbiota; indeed, antibiotic treatment abolishes their therapeutic effects, whereas fecal transplantation restores them (Lin et al., 2024).

*Ginseng* polysaccharides (GPs) are primarily derived from the roots of *Panax ginseng* and are extracted via aqueous or enzymatic methods (Hua et al., 2020). Modern pharmacological studies have demonstrated that GPs possess immunomodulatory, antitumor, antioxidant, and anti-inflammatory effects, including enhanced immune responses via activation of macrophages and T cells (Lee et al., 2021; Li et al., 2016). Studies have shown that GPs significantly reduce intestinal 5-HT levels and inhibit HTR3A receptor signaling by remodeling the gut microbiota—specifically, by enriching *Lactobacillus* and inhibiting pathogenic bacterial proliferation—and subsequently regulating the Trp metabolic pathway. Additionally, GPs effectively maintain intestinal barrier integrity by restoring TJ protein expression and reducing intestinal permeability, while inhibiting the TLR4/MyD88/NF-κB pathway and pro-inflammatory cytokine secretion, ultimately reprogramming the intestinal inflammatory microenvironment. This effect is closely associated with the core pathways through which GPs regulate microbial metabolism and immune homeostasis via multiple targets (Wan L. et al., 2024).

*Panax quinquefolius* polysaccharides (WQP) are the primary active ingredient in the dried roots of *P. quinquefolius* (Guo M. et al., 2021). Modern pharmacological studies have demonstrated that WQP exhibit a range of biological activities, including antitumor, anti-inflammatory, antioxidant, and immunomodulatory effects (Ghosh et al., 2019). WQP have been shown to elevate fecal SCFAs levels by enriching SCFAs-producing genera, and these metabolites exhibit a strong positive correlation with upregulation of TJ protein expression, suggesting that SCFAs—especially butyric acid—may directly promote intestinal barrier repair via activation of the PPAR-γ signaling pathway. Meanwhile, WQP significantly reduce pro-inflammatory factors (IL-1β, TNF-α, IL-6) and induce anti-inflammatory IL-10 secretion by inhibiting the NF-κB pathway, demonstrating a dual immunomodulatory function (Ren et al., 2023).

*Astragalus* polysaccharides (APS), primarily derived from the dried roots of *Astragalus membranaceus*, possess antiviral, anti-inflammatory, and immunomodulatory effects, and enhance macrophage activity by activating the JNK/MAPK, Erk/MAPK, and NF-κB signaling pathways (Li et al., 2015; Aleebrahim-Dehkordi et al., 2022; Lu et al., 2024). APS have been found to significantly increase the abundance of SCFAs-producing bacteria by modulating gut microbiota structure, thereby restoring SCFAs levels and enhancing intestinal barrier function. Meanwhile, SCFAs reduce Th17 differentiation and promote Treg expansion by activating free fatty acid receptor 2/3 (FFAR2/3) and concurrently inhibiting both the TLR4/MyD88/NF-κB pathway and HDAC3-mediated epigenetic regulation (Zhang Y. et al., 2025).

*Poria cocos* polysaccharide (PCP), primarily derived from the dried mycelium of the Polyporaceae fungus *Poria cocos*, exhibits a wide range of biological activities, including antioxidant, immunomodulatory, anti-inflammatory, anticancer, hepatoprotective, and intestinal microbiota–modulating effects (Ng et al., 2024; Lee et al., 2017). In DSS-induced colitis models, PCP improves intestinal barrier function by upregulating TJ proteins such as occludin, claudin-1, and ZO-1. At the molecular level, PCP alleviates intestinal inflammation by inhibiting the NF-κB pathway, reducing pro-inflammatory factor expression (e.g., IL-1β, IL-12, TNF-α), and elevating anti-inflammatory IL-10 levels. Furthermore, PCP modulates intestinal microbiota structure by restoring the abundance of beneficial bacteria and SCFAs production, and its beneficial effects have been validated through fecal microbial transplantation experiments (Wan J. et al., 2024).

*Codonopsis pilosula* polysaccharide (CPP), primarily derived from the root of the traditional Chinese medicine *C. pilosula*, exhibits various pharmacological effects—including immunomodulatory, anti-inflammatory, antioxidant, anti-fatigue, and hepatoprotective activities—and its structural characteristics are closely linked to its biological functions (Ma et al., 2024; Fu et al., 2025; Meng et al., 2020). Recent studies have found that CPP significantly enriches SCFAs-producing probiotic flora (e.g., *Ligilactobacillus* and *Akkermansia*), promotes acetic acid and butyric acid production, and inhibits the NLRP3 inflammasome signaling pathway via activation of its receptor, GPR43/GPR109A, thereby reducing pro-inflammatory factors (e.g., IL-1β and IL-18). Fecal transplantation experiments further validated that the gut microbiota regulated by CPP can effectively alleviate colonic inflammation via the SCFAs-mediated GPR/NLRP3 signaling axis (Zhou et al., 2025).

#### 3.1.2 Alkaloids

Berberine (BBR) is an isoquinoline alkaloid widely distributed in *Berberidaceae* plants such as *Coptis chinensis* and *Phellodendron chinense* (Wang et al., 2019). In UC, BBR may exert therapeutic effects via immune modulation, anti-inflammatory and mucosal repair mechanisms, and regulation of the gut microbiota. Multi-omics data have demonstrated that BBR significantly ameliorates gut microbiota dysbiosis in UC models by restoring metabolic homeostasis and attenuating inflammatory responses—achieved through increasing beneficial bacteria (e.g., *Bacteroides* and *Akkermansia*) and inhibiting arachidonic acid (AA) metabolic pathways (Yang T. et al., 2024). Another study focusing on intestinal neural–immune regulation found that BBR effectively rebuilt the mucosal barrier and reduced immune cell overactivation by preserving intestinal neuroglia function and enhancing interactions between IECs and immune cells, thereby alleviating local inflammation (Li et al., 2020).

Matrine (MT) is a natural quinolizidine alkaloid primarily derived from the legume *Sophora flavescens*, traditionally used to treat skin inflammation, diarrhea, and vaginal itching (Gao et al., 2024). Modern pharmacological studies have demonstrated that MT exhibits multiple biological activities, including anti-inflammatory, antioxidant, immunomodulatory, and gut microbiota–modulating effects (Yao H. et al., 2021; Sun J. et al., 2024). Recent studies have found that MT restores goblet cell numbers and TJ protein expression, suggesting that it alleviates disease progression by repairing intestinal barrier integrity. In addition, MT effectively alleviates oxidative stress injury by upregulating SOD and CAT activities and reducing MDA levels. Notably, MT significantly corrects intestinal immune imbalance, as evidenced by an increased Treg and decreased Th17 proportion in the spleen and mesenteric lymph nodes, suggesting that it inhibits excessive immune responses by regulating the Treg/Th17 balance (Mao et al., 2024).

Evodiamine (EVO) is a quinolone alkaloid isolated from *Evodia rutaecarpa* that exhibits a wide range of biological activities, including anticancer, anti-hypoxia, antithrombotic, anti-inflammatory, and analgesic effects (Yu et al., 2013; Jiang and Hu, 2009). EVO has been found to activate intestinal epithelial GPR43 receptors by promoting acetate production, which in turn inhibits the NF-κB pathway and downregulates pro-inflammatory factor expression. Meanwhile, EVO repairs intestinal barrier function by restoring goblet cell mucin (MUC2) secretion, upregulating Reg3γ/β antimicrobial peptides, and enhancing TJ protein Claudin-1 expression. Fecal microbiota transplantation experiments further confirmed that EVO-modulated microbiota exert independent therapeutic effects, while *Lactobacillus* supplementation partially mimics EVO’s anti-inflammatory effects, highlighting the central role of microbiota metabolites (Wang et al., 2020).

Palmatine (PAL) is a natural isoquinoline alkaloid primarily derived from traditional Chinese medicinal plants such as *C. chinensis* and *P. chinense* (Ji et al., 2024a). Studies have shown that PAL ameliorates clinical symptoms and histopathological changes in DSS-induced UC models by inhibiting pro-inflammatory factor secretion (e.g., TNF-α, IL-1β, IL-6, IL-18), reducing oxidative stress and iron loading, and modulating the expression of GPX4, ACSL4, COX-2, and Nrf2/HO-1 (Ji et al., 2024b). Another study found that 8-Oxypalmatine (OPAL), a metabolite of PAL *in vivo*, exhibits superior therapeutic effects. OPAL not only repairs the intestinal mucus barrier and restores TJ protein expression but also effectively alleviates UC inflammatory responses by activating AMPK, inhibiting the NF-κB pathway, and promoting macrophage polarization from M1 to M2 (Huang et al., 2025).

Sinomenine, an isoquinoline alkaloid, is primarily extracted from the dried vine stems of *Sinomenii Caulis*. Modern pharmacological studies have demonstrated that sinomenine possesses significant anti-inflammatory, immunosuppressive, and antitumor activities, achieving its effects by inhibiting inflammatory factor release and modulating immune cell function, particularly in UC treatment (Zhang et al., 2024b; Tian et al., 2024). Further experiments showed that sinomenine activates the HO-1/Nrf2 pathway, upregulates SOD and CAT activities, and simultaneously inhibits RIP1 and Caspase-1 mRNA expression—key components of the NLRP3 inflammasome—to achieve a dual blockade of oxidative stress and inflammatory cascades. Particularly, 16S rRNA sequencing confirmed that sinomenine can reconfigure the gut microbiota, resulting in an increased *Firmicutes*/*Bacteroidetes* (F/B) ratio and higher abundance of probiotics (e.g., *Lactobacillus spp*), thereby enhancing intestinal barrier function via colony–immunity interactions (Niu et al., 2024).

#### 3.1.3 Flavonoids and Polyphenols

Galangin, a natural flavonoid primarily derived from *Alpinia officinarum*, exhibits anti-inflammatory, antioxidant, and immunomodulatory effects. Galangin has been found to activate the autophagy pathway, significantly upregulate ATG5, ATG7, and the LC3B-II/I ratio, promote clearance of damaged cells, and inhibit activation of NLRP3 inflammasomes. In addition, Galangin remodels gut microbiota structure, enhances α-diversity, and enriches butyric acid–producing bacteria—thereby driving butyric acid production that enhances intestinal barrier function and inhibits NF-κB–mediated release of TNF-α and IL-1β via PPAR-γ signaling (Xuan et al., 2020). Another study found that Galangin targets the TLR4/NF-κB pathway, downregulates TLR4 mRNA and HMGB1 expression, and reduces NF-κB nuclear translocation, thereby decreasing pro-inflammatory factor levels. Its phenolic hydroxyl groups directly scavenge ROS and enhance antioxidant enzyme activities, synergistically mitigating oxidative damage. However, the long-term efficacy, human bioavailability, and capacity to modulate intestinal barrier proteins of Galangin remain to be thoroughly verified (Gerges et al., 2020).

Baicalin, a flavonoid extracted from *Scutellaria baicalensis*, exhibits a wide range of activities, including anti-inflammatory, antioxidant, immunomodulatory, and anti-apoptotic effects (Jia Y. et al., 2024; Zou et al., 2015; Hu et al., 2021). Studies have shown that Baicalin significantly corrects Th17/Treg imbalance and repairs the intestinal barrier by upregulating tight junction proteins and mucin MUC2. Meanwhile, Baicalin elevates fecal butyric acid levels by enriching butyric acid–producing bacteria and activates the butyric acid–GPR43/HDAC inhibitory axis to enhance anti-inflammatory effects (Zhu et al., 2020). Zhang et al. developed water-soluble baicalin magnesium (BA-Mg), which significantly increases secondary bile acid levels, activates FXR/GPBAR1 receptors, and inhibits the NF-κB inflammatory pathway. Moreover, BA-Mg promotes the proliferation of bile acid–converting bacteria (e.g., *Oscillibacter*) by remodeling the microbiota structure, thereby forming a positive feedback loop among flora, BAs, and immunity (Zhang L. et al., 2024).

Alpinetin, a flavonoid extracted from the seeds of *Alpinia katsumadai*, exhibits a wide range of biological activities (Lv et al., 2018). In a DSS-induced colitis model, Alpinetin significantly attenuates intestinal inflammatory injury by restoring colonic Th17/Treg balance. Further studies revealed that Alpinetin promotes miR-302 expression in CD4^+^ T cells via activation of the AhR/ARNT/XRE pathway while inhibiting DNMT-1 activity, thereby reducing Foxp3 promoter methylation and enhancing Treg cell differentiation and function. In addition, Alpinetin specifically enhances CREB binding to the Foxp3 promoter without affecting CREB nuclear translocation or DNA binding, suggesting that it strengthens Treg cell immunosuppressive function via epigenetic reprogramming (Lv et al., 2018).

Curcumin (Cur) is a natural polyphenolic compound extracted from the rhizome of *Curcuma longa*, a ginger family plant, and is one of the active ingredients of TCM. Modern pharmacological studies have shown that Cur exhibits significant anti-inflammatory, antioxidant, antitumor, and immunomodulatory effects (Nelson et al., 2017). In UC treatment, Cur exerts protective effects via multiple pathways. At the immunomodulatory level, Cur bidirectionally regulates the adaptive immune system: on the one hand, it promotes Treg cell differentiation via TGF-β1/Smad3 pathway activation and inhibits Th17 cell polarization to restore Treg/Th17 homeostasis (Guo et al., 2022); on the other hand, its inhibition of the TLR4/MyD88/NF-κB axis through epigenetic regulation reduces PD-L1^+^ inhibitory B cells while promoting IL-10 secretion in CD19^+^CD1d^+^CD5^+^ B10 cells, thereby enabling cross-talk among T cells, B cells, and epithelial cells (Huang et al., 2023). In terms of intrinsic immune defense, Cur directly targets RIP3—a key regulator of necroptosis in IECs—and blocks formation of the RIP1-RIP3-MLKL complex by occupying its RHIM domain, thereby reducing phosphorylated MLKL levels and the release of intestinal barrier DAMPs (Y et al., 2023).

#### 3.1.4 Terpenoids and glycosides

Andrographolide (AND) is a diterpenoid isolated from *Andrographis paniculata*, a plant in the *Jurassicaceae* family, and exhibits a wide range of biological effects as its main active ingredient (Jing et al., 2019). Zhu et al. found, based on *in vitro* experiments with peripheral blood mononuclear cells (PBMCs) from UC patients, that AND remodels T cell subset balance by downregulating the Th1-specific transcription factor T-bet and the Th17 key regulator ROR-γt, while upregulating the Th2 marker GATA-3. AND is particularly effective at reversing IL-23-induced hyperpolarization of Th1/Th17, suggesting that its targeted inhibition of the IL-23/Th17 inflammatory axis represents a central mechanism for ameliorating immune imbalance in UC (Zhu et al., 2018). Further studies revealed that AND significantly alleviates oxazolone-induced intestinal inflammation in a rat UC model by modulating the IL-4R/STAT6 pathway—specifically inhibiting STAT6 phosphorylation while enhancing IL-4 receptor expression—suggesting that it exerts anti-inflammatory effects through dual modulation of the Th2-related pathway (Zhang et al., 2019).

Triptolide is a diterpene lactone active compound isolated from *Tripterygium wilfordii*, a traditional Chinese medicine, and exhibits remarkable anti-inflammatory, immunomodulatory, and antitumor properties (Tang et al., 2020; Moudgil and Venkatesha, 2022). Triptolide has been found to inhibit macrophage polarization toward the pro-inflammatory M1 phenotype and reduce ROS production via activation of the NRF2/HO-1 antioxidant pathway, thereby attenuating oxidative stress damage. In addition, Triptolide inhibits the PDE4B-dependent AKT/NF-κB signaling cascade, thereby downregulating pro-inflammatory cytokine expression (e.g., TNF-α, IL-6) and blocking the amplification of intestinal inflammation (Tang et al., 2020). Another study addressed the toxicity bottleneck of Triptolide by designing a novel derivative, ZT01, to reduce its reproductive toxicity via structural modification. Experiments showed that ZT01 treatment significantly improves intestinal epithelial barrier function, reduces Th1 and Th17 cell differentiation, and regulates JAK-STAT signaling activity, suggesting its therapeutic role by inhibiting T-cell-mediated immune abnormalities and macrophage-driven inflammatory responses (Fu et al., 2021).

Glycyrrhizin is a triterpenoid saponin compound primarily derived from the rhizomes of *Glycyrrhiza glabra*, a leguminous plant, and is one of its most abundant active ingredients. Modern pharmacological studies have shown that Glycyrrhizin exhibits significant anti-inflammatory, antioxidant, and immunomodulatory effects. Animal experiments revealed that Glycyrrhizin significantly alleviates DSS-induced histopathological damage in the mouse colon, reduces overproduction of IL-6 and IL-17 in the colonic mucosa, and demonstrates high-affinity binding to inflammation-related targets (e.g., TLR4/MyD88 pathway proteins) as confirmed by molecular docking analysis. Meanwhile, Glycyrrhizin inhibits LPS-induced inflammatory injury in IECs in cellular models, suggesting its therapeutic role in maintaining intestinal barrier integrity (Kong et al., 2024).

Tanshinone IIA (Tan IIA) is a major fat-soluble diterpene quinone extracted from the roots and rhizomes of *Salvia miltiorrhiza*, a traditional Chinese medicinal herb, and exhibits a wide range of modern pharmacological activities (Zhang X. et al., 2015). In UC treatment, Tan IIA significantly ameliorates histopathological damage in the colon of experimental animals, reduces the disease activity index score, and modulates serum inflammatory factor levels—lowering pro-inflammatory factors (TNF-α, IL-1β, and IL-6) while elevating anti-inflammatory IL-10 (Zhu et al., 2022). In addition, its mechanism may involve inhibition of the TLR4/PI3K/AKT/mTOR pathway, thereby reducing apoptosis and inflammatory cell infiltration in IECs (Peng et al., 2021). Clinical studies have shown that Tan IIA combined with mesalazine significantly increases overall treatment efficacy and decreases TNF-α and CRP levels in UC patients, suggesting its potential as an adjuvant therapy (Chen et al., 2024c). However, its long-term efficacy and safety remain to be further validated in multicenter clinical studies.

Salidroside (SAL) is a glycoside compound extracted from the perennial herb *Rhodiola rosea* and exhibits anticancer, anti-inflammatory, and antioxidant activities (Zhang et al., 2021). Recent studies have found that SAL inhibits NLRP3 inflammasome activation by targeting the TREM1/SYK signaling axis, as evidenced by reduced expression of key proteins (NLRP3, caspase-1 p20, and GSDMD p30) in colonic tissues, and that TREM1 knockdown completely blocks its regulatory effect on NLRP3. Notably, SAL significantly remodels gut microbiota structure, resulting in an elevated *Firmicutes*/*Bacteroidetes* (F/B) ratio and specific enrichment of SCFAs-producing genera. The microbiota-dependent restoration of Th17/Treg balance was confirmed by antibiotic clearance and fecal transplantation experiments (Liu X. et al., 2023).

Paeoniflorin (PF) is a monoterpene glycoside primarily derived from the dried roots of *Paeonia lactiflora* and *Paeonia veitchii*, exhibiting anti-inflammatory, antioxidant, immunomodulatory, and neuroprotective effects (Li Q. et al., 2024). PF has been found to reduce the abnormal accumulation of the Trp metabolite indole-3-lactic acid (ILA) by modulating intestinal microbiota structure. As a key effector, ILA inhibits autophagy in junctional IECs by activating mTOR signaling, thereby enhancing NLRP3 inflammasome activation. In contrast, PF treatment significantly restores autophagic flux and blocks the release of pro-inflammatory factors (e.g., IL-1β), thereby alleviating DSS-induced colitis (Fan et al., 2020). Another *in vitro* experiment showed that PF-pretreated dendritic cells significantly promote Treg (Foxp3^+^IL-10^+^) differentiation and inhibit Th17 (IL-17A^+^) polarization, reducing the Th17/Treg ratio by over 80%. In a TNBS colitis model, PF restores intestinal immune tolerance by regulating T cell differentiation through inhibition of dendritic cell maturation (Zheng et al., 2020). Further studies have found that PF also exhibits significant mucosal regenerative and reparative functions; it promotes the proliferation and directed differentiation of ISCs by activating the PI3K-AKT-mTOR pathway, thereby driving regeneration of goblet and Paneth cells and accelerating epithelial barrier repair (Ma et al., 2023).

Ginsenosides are a class of triterpenoid saponins extracted from *P. ginseng*, a member of the *Araliaceae* family, and constitute the main active ingredients of ginseng (Yuan et al., 2024; Zhou et al., 2024b). Based on structural differences, ginsenosides can be categorized into proto-ginseng diol types (e.g., Rb1, Rd, Rh2) and proto-ginseng triol types (e.g., Rg1, RT4), with their biosynthesis involving glycosyltransferase-mediated modifications (Li Y. et al., 2023; Guan and Qi, 2023). In UC treatment, several ginsenosides exert therapeutic effects through multi-target mechanisms. Ginsenoside Rb1 (GRb1) attenuates DSS-induced ulcerative colitis in mice by synergistically modulating the VDR/PPARγ/NF-κB pathway—inhibiting TNF-α and IL-6 while upregulating IL-10 expression—and repairs the intestinal barrier by enhancing tight junction proteins (e.g., ZO-1, occludin, and E-cadherin), as validated by multi-omics analysis and molecular docking (Zhou et al., 2024b). Ginsenoside Rd (GRd) induces p62-mediated mitochondrial autophagy by activating the AMPK/ULK1 pathway, removes damaged mitochondria, and inhibits NLRP3 inflammasome activation, thereby attenuating DSS-induced colitis in mice (Liu et al., 2018). In addition, in a rat TNBS model of recurrent colitis, GRd effectively reduces levels of the oxidative stress marker MDA and pro-inflammatory mediators (iNOS/NO) by inhibiting neutrophil infiltration (decreasing MPO activity), modulating Ca^2+^ signaling, and augmenting SOD/GSH-Px enzyme activity (Yang et al., 2012). Ginsenoside Rh2 (GRh2) significantly enhances SMAD2/3 phosphorylation and inhibits pro-inflammatory factors (IL-6, TNFα, IFNγ) via activation of the TGFβ signaling pathway, with its anti-inflammatory effects reversed by the TGFβ receptor I inhibitor SB431542 (Ye et al., 2014). Ginsenoside Rg1 (GRg1) repairs the intestinal barrier by upregulating the TJ protein ZO-1, reducing pro-inflammatory factors (TNF-α and IFN-γ), and elevating anti-inflammatory IL-4 levels, with efficacy comparable to the first-line drug 5-ASA (He et al., 2022). In a DSS-induced mouse model of colitis, Ginsenoside RT4 (GRT4) inhibits its target gene SLC7A11 by upregulating miR-144-3p expression, reduces pro-inflammatory factor expression (e.g., TNF-α and IL-1β), and elevates anti-inflammatory IL-10 levels. In addition, GRT4 promotes macrophage polarization from the pro-inflammatory M1 to the anti-inflammatory M2 phenotype by regulating the miR-144-3p/SLC7A11 pathway, thereby accelerating colonic tissue repair (Li Y. et al., 2023) (Table 1).

**TABLE 1:** Summary of TCM active ingredients in the treatment of UC.

Name	Source	Associated mechanisms	Reported targets	References
Polysaccharides				
*Polygonatum cyrtonema* polysaccharides	*Polygonatum cyrtonema*	Regulation of inflammatory factors Antioxidant activity Repair of intestinal barrier Regulation of gut microbiota	IL-6, IL-1β, TNF-α↓, IL-10↑ MDA↓, SOD, GSH↑ ZO-1, occludin, claudin↑ *Akkermansia muciniphila*, *Muribaculum*↑, UBA3263、CAG-873↓	Lin et al. (2024)
*Ginseng* polysaccharides	*Panax ginseng*	Regulation of inflammatory factors Repair of intestinal barrier Regulation of gut microbiota Regulation of Trp metabolism and 5-HT/HTR3A pathway	IL-6, IL-1β, TNF-α, MCP-1, GM-CSF, IFN-γ↓, IL-10↑ ZO-1, occludin, claudin-1↑ *Lactobacillus*↑, *Escherichia-Shigella*↓ IPA↑, HTR3A, 5-HT↓	Wan et al. (2024b)
*Panax quinquefolius* polysaccharides	*Panax quinquefolius*	Regulation of inflammatory factors Regulation of gut microbiota and its metabolites Repair of intestinal barrier	IL-6, IL-8, IL-1β, TNF-α↓, IL-4, IL-10↑ *Bacteroidetes*, *Rikenellaceae*↑, *Firmicutes*, *Lactobacillus*↓, SCFAs↑ ZO-1, occludin, claudin-1↑	Ren et al. (2023)
*Astragalus* polysaccharides	*Astragalus membranaceus*	Regulation of gut microbiota and its metabolites Repair of intestinal barrier Modulation of inflammatory pathways Restoration of Th17/Treg balance	*Muribaculaceae*, *Lachnospiraceae*↑, *Bacteroides*, *Enterobacteriaceae*↓, SCFAs↑, FFAR2/FFAR3 ↑; claudin-1, Occludin, ZO-1↑, LPS↓ TLR4/MyD88/p-IκBα↓, HDAC3↓, IL-6/p-JAK2/p-STAT3↓ IL-17A^+^CD4^+^↓, CD25^+^Foxp3^+^↑	Zhang et al. (2025b)
*Poria cocos* polysaccharide	*Poria cocos*	Repair of intestinal barrier Inhibition of NF-κB pathway Regulation of inflammatory factors Regulation of gut microbiota and its metabolites	Occluding, claudin-1, ZO-1↑, DAO↓; p-IκBα, p-NF-κB p65↓ IL-1β, IL-12, TNF-α↓, IL-10↑ *Akkermansiaceae*, *Lactobacillaceae*↑, *Erysipelotrichaceae*↓, SCFAs↑	Wan et al. (2024c)
*Codonopsis pilosula* polysaccharide	*Codonopsis pilosula*	Regulation of gut microbiota and its metabolites Regulation of GPR/NLRP3 pathway Regulation of inflammatory factors	*Ligilactobacillus*, *Akkermansia*↑, *Duncaniella*↓, Acetate, Butyrate↑ GPR43/GPR109A↑, NLRP3/caspase-1↓ IL-1β, IL-6, TNF-α↓	Zhou et al. (2025)
Alkaloids				
Berberine	*Coptis chinensis* *Phellodendron chinense*	Regulation of gut microbiota Inhibition of AA metabolism	*Lactobacillus*, *Escherichia-Shigella*↓, *f_Muribaculaceae, Bacteroides*, *Akkermansia*↑ LTA4, LA, α-LA↓, TXB2↑	Yang et al. (2024b)
		Regulation of inflammatory factors Antioxidant activity Repair of intestinal barrier Regulating the function of EGCs Inhibition of immune cell activation and infiltration	TNF-α, IL-1β, IL-17A, IL-6↓, IL-10↑ MDA, MPO↓, SOD↑ Claudin-1, ZO-1, E-cadherin↑; substance P, BDNF↓, GDNF, GFAP↑ CD11b^+^F4/80^+^, CD11b^+^Gr-1^+^, CD11b^+^CD11c^+^↓, Th1(IFN-γ^+^), Th17(IL-17A^+^)↓	Li et al. (2020)
Matrine	*Sophora flavescens*	Regulation of inflammatory factors Antioxidant activity Repair of intestinal barrier Restoration of Th17/Treg balance Regulation of gut microbiota	TNF-α, IL-1β, IL-17A, IL-6↓, IL-10↑ MDA↓, SOD, CAT↑ ZO-1, Occludin↑ Th17(CD4^+^IL-17A^+^)↓, Treg(CD4^+^Foxp3^+^)↑ *Firmicutes*/*Bacteroidetes*, *Proteobacteria*↓, *Lactobacillus*, *Akkermansia*↑	Mao et al. (2024)
Evodiamine	*Evodia rutaecarpa*	Regulation of gut microbiota and its metabolites Repair of intestinal barrier Regulation of inflammatory factors	*Lactobacillus acidophilus*↑ *Mucispirillum*, *Parabacteroides*↓, Acetate↑ Claudin-1, Occludin, MUC2↑ TNF-α, IL-1β, IL-17A, IL-6↓, IL-10↑	Wang et al. (2020)
Palmatine	*Coptis chinensis* *Phellodendron chinense*	Regulation of inflammatory factors Antioxidant activity Inhibition of ferroptosis	TNF-α, IL-1β, IL-18, IL-6↓ GSH↑, MDA, NO, LDH↓ GPX4, p-Nrf2↑, ACSL4, COX-2, HO-1↓	Ji et al. (2024b)
Sinomenine	*Sinomenii Caulis*	Antioxidant activity Inhibition of NLRP3 inflammasome activation Regulation of inflammatory factors Regulation of gut microbiota	SOD, CAT, GPx, GR↑, MDA↓ NLRP3, caspase-1, IL-18↓ TNF-α, IL-1β, IL-6, IL-17↓, IL-10↑ *Firmicutes/Bacteroidetes*↓, *Akkermansia*↑	Niu et al. (2024)
Flavonoids and Polyphenols				
Galangin	*Alpinia officinarum*	Activation of autophagy Regulation of gut microbiota and its metabolites Regulation of inflammatory factors	ATG5, ATG7, ATG12, LC3B-II/I↑ *Lactobacillus*, *Butyricimonas*↑, *Escherichia-Shigella*↓, SCFAs↑ TNF-α, IL-1β, IL-6, MPO↓	Xuan et al. (2020)
		Inhibition of TLR4/NF-κB pathway Regulation of inflammatory factors Antioxidant activity	TLR4, NF-κB p65↓ IL-6, TNF-α, HMGB1↓ GSH↑, MDA, LDH↓	Gerges et al. (2020)
Baicalin	*Scutellaria baicalensis*	Restoration of Th17/Treg balance Repair of intestinal barrier Antioxidant activity Regulation of gut microbiota and its metabolites	Th17↓, Treg↑ ZO-1, Occludin, MUC2↑ MDA↓, GSH, SOD↑ *Butyricimonas*↑, *Proteobacteria*↓, SCFAs↑	Zhu et al. (2020)
		Regulation of gut microbiota and its metabolites Regulation of NF-κB/PPAR-α pathway Regulation of inflammatory factors Antioxidant activity Repair of intestinal barrier	*Bacteroidota*/*Firmicutes*, *Oscillibacter*↑, *Bacteroides*, *Escherichia*↓, β-MCA, ACA↓, DCA, UDCA, CDCA↑; p-IκB, p-P65↓, PPAR-α↑ TNF-α, IL-1β, IL-6↓, IL-10↑ MDA↓, SOD↑ Occludin, ZO-1, Claudin-1↑	Zhang et al. (2024c)
Alpinetin	*Alpinia katsumadai*	Activation of AhR pathway Regulation of miR-302/DNMT-1 pathway Restoration of Th17/Treg balance Regulation of inflammatory factors	CYP1A1↑; miR-302↑, DNMT-1↓ RORγt↓, Foxp3↑ TNF-α, IL-1β, IL-17↓, IL-10↑	Lv et al. (2018)
Curcumin	*Curcuma longa*	Restoration of Th17/Treg balance Regulation of inflammatory factors	CD4^+^CD25^+^Foxp3^+^↑, CD4^+^IL17^+^↓ IL-17A↓, IL-10↑	Guo et al. (2022)
		Regulation of Breg subsets Inhibition of TLR/MyD88 pathway Regulation of inflammatory factors	CD1d^+^, CD25^+^, Foxp3^+^↑, PD-L1^+^, Tim-1^+^, CD27^+^↓ TLR2, TLR4, TLR5, MyD88, p-IRAK4, NF-κB p65, IRAK1, p38 MAPK↓ IL-1β, IL-6, IL-33, CCL-2, IFN-γ, TNF-α↓, IL-4, IL-10, IL-13↑	Huang et al. (2023)
Terpenoids and Glycosides				
Andrographolide	*Andrographis paniculata*	Regulation of Th cell homeostasis Regulation of inflammatory factors	T-bet, RORγt↓, GATA-3↑ IFN-γ, IL-23, IL-17A↓, IL-4↑	Zhang et al. (2019)
Triptolide	*Tripterygium wilfordii*	Inhibition of macrophage M1 polarization Antioxidant activity Inhibition of PDE4B/AKT/NF-κB axis Repair of intestinal barrier Regulation of inflammatory factors	CD86, CD80↓, CD206, ARG-1↑ ROS↓, NRF2, HO-1, GSS, GCLM↑ PDE4B, p-AKT, p-P65↓ Claudin-1, Occludin↑ IL-1β, IL-6, TNF-α↓	Tang et al. (2020)
Glycyrrhizin	*Glycyrrhiza glabra*	Antioxidant activity Regulation of inflammatory factors Induction of mitochondrial autophagy	Nrf2, HO-1↑, SOD, GSH-PX↑, MDA↓ IL-1β, IL-6, TNF-α, IL-17↓, IL-10↑ PINK1, Parkin↑, LC3-II/LC3-I↑, P62↓	Kong et al. (2024)
Tanshinone IIA	*Salvia miltiorrhiza*	Regulation of metabolism Regulation of inflammatory factors	Taurine, L-Glutamine, Hippuric Acid↑, 8-HETE, LysoPC/LysoPE↓ IL-1β, IL-6, TNF-α↓, IL-10↑	Zhu et al. (2022)
Salidroside	*Rhodiola Rosea*	Inhibition of macrophage pyroptosis Regulation of gut microbiota Restoration of Th17/Treg balance Regulation of inflammatory factors	NLRP3, Caspase-1, GSDMD p30, TREM1↓ *Firmicutes*↑, *Bacteroidetes*, *Akkermansia*↓ Th17↓, Treg↑ IL-1β, IL-6, TNF-α, IFN-γ↓, IL-10↑	Liu et al. (2023b)
Paeoniflorin	*Paeonia lactiflora* *Paeonia veitchii*	Regulation of gut microbiota and its metabolites Activation of epithelial cell autophagy Regulation of inflammatory factors	*Firmicutes*, *Lactobacillus*↑, *Verrucomicrobia*↓, ILA, IAA↓ LC3-II/I↑, P62↓ IL-1β, IL-6, IL-17↓	Fan et al. (2020)
		Inhibition of DCs maturation Restoration of Th17/Treg balance	MHC-II^+^CD86^+^ DCs, IL-12↓ Th17, IL-17↓, Treg, IL-10, Foxp3↑	Zheng et al. (2020)
Ginsenoside Rb1	*Panax ginseng* Meyer	Inhibition of NF-κB pathway Regulation of inflammatory factors Repair of intestinal barrier	PPARγ, VDR↑, p-NF-κB p65↓ IL-6, TNF-α↓, IL-10↑ ZO-1, Occludin, E-cadherin↑	Zhou et al. (2024b)
Ginsenoside Rd	*Panax ginseng* Meyer	Induction of mitochondrial autophagy Inhibition of NLRP3 inflammasome Regulation of inflammatory factors Antioxidant activity	AMPK, ULK1, p62, LC3-II/LC3-I↑ NLRP3, caspase-1, ASC↓ IL-1β, TNF-α, IL-6↓ MPO, iNOS↓, GSH↑	Liu et al. (2018)
Ginsenoside Rh2	*Panax ginseng* Meyer	Activation of TGFβ/SMAD pathway Regulation of inflammatory factors	pTGFβ receptor I, pSMAD2, pSMAD3↑ IL-6, TNF-α, IFN-γ↓	Ye et al. (2014)
Ginsenoside Rg1	*Panax ginseng* Meyer	Repair of intestinal barrier Regulation of inflammatory factors	ZO-1↑ IFN-γ, TNF-α↓, IL-4↑	He et al. (2022)
Ginsenoside RT4	*Panax ginseng* Meyer	Modulation of miR-144-3p/SLC7A11 axis Regulation of inflammatory factors	miR-144-3p↑, SLC7A11↓ IL-1β, TNF-α↓, IL-10↑	Li et al. (2023b)

The symbol “↓” indicates inhibition, while “↑” represents promotion.

### 3.2 TCM herbal formulas

Shenling Baizhu Powder (SBP) is a traditional Chinese herbal formula known for strengthening the spleen, benefiting qi, resolving dampness, and stopping diarrhea, and is commonly used to treat digestive disorders of the spleen-deficiency and dampness-abundance type (Chen et al., 2022). SBP has been found to repair the damaged intestinal barrier in TNBS-induced rat colitis by restoring normal expression and distribution of intestinal epithelial TJ proteins and promoting production of the key mucus component, MUC2. In addition, SBP significantly inhibits apoptosis, as evidenced by a reduced Bax/Bcl-2 ratio and lower caspase-3 activity. Further mechanistic studies have shown that SBP regulates the TLR5/MyD88/NF-κB pathway and reduces inflammatory signaling, thereby slowing the inflammatory response (Rao et al., 2022).

Sishen Pills (SSP) is a classic Chinese compound used for treating chronic diarrhea and other digestive disorders associated with spleen and kidney yang deficiency (Tao et al., 2022). By integrating transcriptome sequencing and network pharmacology analysis, SSP was found to significantly downregulate miR-505-3p in colon tissues, thereby inhibiting CDH1-encoded E-cadherin expression and blocking dendritic cell differentiation into a pro-inflammatory phenotype. *In vitro* and *in vivo* experiments confirmed that this mechanism effectively reduces secretion of pro-inflammatory factors (IL-6, IL-1β, TNF-α) and ameliorates DSS-induced colonic injury. Furthermore, SSP studies identified EVO as a key active component via molecular docking and functional validation, showing that it exerts anti-inflammatory effects through the same pathway (Huang J. et al., 2024).

Shaoyao Decoction (SYD) is a traditional Chinese formula known for clearing heat, resolving dampness and dryness, and regulating qi and blood, and is commonly used to treat damp-heat diarrhea. In terms of immunomodulation, SYD significantly reduces the proportion of Th17 cells and RORγt expression by inhibiting the IL-6/STAT3 axis, while upregulating Treg cell numbers and Foxp3 levels to restore the Th17/Treg balance. This immune remodeling effect is accompanied by a decrease in pro-inflammatory factors (IL-17, IL-6) and an increase in anti-inflammatory factors (IL-10, TGF-β1) expression (Wu D. et al., 2023). In addition, SYD remodels gut microbiota by increasing the abundance of butyric acid–producing bacteria and promoting SCFAs production, while regulating Trp metabolism and restoring indole metabolite levels (IA, IAA, IAld) to normal. These metabolites induce IL-22 secretion via AHR receptor activation, which in turn activates STAT3 phosphorylation and promotes expression of intestinal epithelial Reg3β/γ antimicrobial peptides and repair of TJ proteins ZO-1 and occludin (Zhang Y. et al., 2024).

Sijunzi Decoction (SJZD) is a classic tonic formula of TCM, originating from the “Taiping Huimin Heji Prescription” of the Song Dynasty and serving as a core treatment for Spleen-Qi deficiency. SJZD restores gut microbiota homeostasis by reducing the abundance of Proteobacteria and Escherichia-Shigella. In addition, SJZD promotes BAs synthesis—especially taurodeoxycholic acid (TUDCA), a metabolite that improves intestinal barrier function and inhibits inflammatory responses via the TGR5-cAMP pathway—thus exerting anti-UC effects (Wu Y. et al., 2023). Another study further supported the anti-inflammatory and barrier-protective effects of SJZD, finding that it significantly reduces inflammatory factor levels (e.g., IL-6, IL-1β, TNF-α) and upregulates key TJ proteins, such as occludin and ZO-1, while demonstrating that SJZD alleviates UC by remodeling gut microbiota (e.g., modulating levels of *Alistipes*, *Akkermansia*, and *Lachnospiraceae*) and revealing correlations among gut microbiota, inflammatory factors, and TJ proteins (Li H. et al., 2024).

Lizhong Decoction (LZD) is a classic warming formula in TCM, originating from Zhang Zhongjing’s “Treatise on Miscellaneous Diseases of Typhoid” during the Eastern Han Dynasty, and represents a classic prescription for treating cold deficiency of the spleen and stomach. Studies have demonstrated that LZD inhibits the secretion of pro-inflammatory factors (TNF-α, IL-6, IFN-γ), enhances the expression of anti-inflammatory factors (IL-4, IL-10), and modulates the inflammatory cascade by downregulating the TLR4/NF-κB signaling pathway. Notably, LZD also significantly upregulates the expression of TJ proteins (ZO-1, occludin, and claudin-1) and repairs the intestinal mechanical barrier function (Shen et al., 2020). Gut microbiota-based studies further revealed that LZD modifies the gut microbiota structure in UC mice by reducing the abundance of conditionally pathogenic bacteria (e.g., *Clostridium sensu stricto 1*, *Escherichia-Shigella*) and promoting the proliferation of SCFAs-producing flora (e.g., *Blautia*, *Prevotellaceae UCG-001*), thereby improving intestinal microenvironmental homeostasis through the regulation of purine metabolism, BAs synthesis, and α-linolenic acid metabolism, among other pathways (Zou et al., 2020).

Huangqin Decoction (HQD) is a TCM compound primarily used to treat diarrhea, dysentery, and damp-heat conditions, with demonstrated efficacy in clearing heat, removing toxins, harmonizing the middle, and relieving pain. In a mouse model of DSS-induced colitis, HQD enhanced the proliferative capacity of IECs by remodeling gut microbiota homeostasis (evidenced by an increased F/B ratio), elevating the levels of 12 amino acid metabolites (including L-leucine), and activating the mTOR pathway to promote S6/4E-BP1 phosphorylation. Additionally, HQD upregulated the expression of TJ proteins (ZO-1, occludin, and claudin-1) and the adhesion junction protein E-cadherin, thereby repairing intestinal mucosal barrier function. *In vitro* experiments further confirmed that microbiota metabolites and key amino acids regulated by HQD inhibited DSS-induced apoptosis in FHC cells and reduced the expression of pro-apoptotic proteins Bax and cleaved caspase-3, while preserving the integrity of intercellular junctions (Li M-X. et al., 2022).

Dahuang Mudan Decoction (DMD), derived from Zhang Zhongjing’s “The Essentials of the Golden Chamber” during the Eastern Han Dynasty, is a classic TCM formula for treating intestinal carbuncle, exhibiting effects such as relieving diarrhea and heat, breaking up stasis, dispersing knots, and eliminating swelling. Studies have shown that DMD effectively reduces intestinal permeability and serum endotoxin levels by restoring the expression of colonic epithelial TJ proteins (ZO-1, occludin, and claudin-1), thereby limiting the translocation of pathogenic bacteria from the intestinal mucosa to peripheral immune organs. Further immunological analyses revealed that DMD modulates the subpopulation ratio of ILC3 cells in the intestine by increasing NCR^+^ ILC3 cells, which secrete the repair factor IL-22, and by inhibiting NCR^−^ ILC3 cells, which secrete inflammatory factors such as IL-17A and GM-CSF. *In vitro* experiments also confirmed that conditioned medium from DMD-treated cells promoted Caco-2 cell migration and enhanced TJ protein expression, thereby strengthening epithelial barrier function (Huang et al., 2022).

Banxia Xiexin Decoction (BXD) originates from Zhang Zhongjing’s “Treatise on Typhoid Miscellaneous Diseases” during the Eastern Han Dynasty, and modern studies have confirmed its multi-targeted effects on regulating gastrointestinal function. A recent study found that BXD upregulates the expression of TJ proteins (ZO-1 and occludin) in the colon, thereby repairing the damaged intestinal barrier. In addition, BXD modulates the composition and diversity of the intestinal microbiota by significantly increasing the abundance of beneficial bacteria (e.g., *Muribaculaceae*, *Akkermansia*, and *Lactobacillus*) and inhibiting inflammation-associated flora (e.g., *Faecalibaculum* and *Alloprevotella*), thereby improving key metabolic pathways, including those involving SCFAs. Fecal transplantation experiments further verified that microbiota remodeling plays a key role in its anti-UC effects. Overall, BXD achieves combined anti-inflammatory and mucosal repair effects by restoring intestinal microecological balance and regulating the metabolic network (Luo et al., 2024).

Baitouweng Decoction (BD) is a TCM compound with a long history of clinical application, and modern studies have confirmed its favorable therapeutic effect in treating UC (Li X. et al., 2023). Studies have shown that BD reduces pro-inflammatory factors (e.g., IL-1β, IL-6, and TNF-α) while elevating the expression of the anti-inflammatory factor IL-10 by inhibiting Th17 cell differentiation and promoting Treg cell expansion. Regarding intestinal barrier protection, BD significantly reduces intestinal permeability by inhibiting the phosphorylation of the ERK/NF-κB signaling pathway and promoting the synthesis of TJ proteins, including occludin and ZO-1 (Miao et al., 2020). Further studies found that BD remodels the intestinal microbiota, resulting in a decreased abundance of Proteobacteria, elevated production of SCFAs, and modulated mucosal immunity via inhibition of the IL-6/STAT3 pathway (Chen et al., 2021) (Table 2).

**TABLE 2:** Summary of TCM herbal formulas in the treatment of UC.

Name	Components	Associated mechanisms	Reported targets	References
Shenling Baizhu Powder	*Atractylodes macrocephala* Koidz. (Bai Zhu), *Poria cocos* (Schw.) Wolf (Fu Ling), *Glycyrrhiza uralensis* Fisch. (Gan Cao), *Platycodon grandiflorum* (Jacq.) A. DC. (Jie Geng), *Panax ginseng* C.A.Mey. (Ren Shen), *Amomum villosum* Lour. (Sha Ren), *Dioscorea opposita* Thunb. (Shan Yao), *Coix lacryma-jobi* L. var. *mayuen* (Roman.) Stapf (Yi Yi Ren), *Nelumbo nucifera* Gaertn. (Lian Zi) and *Dolichos lablab* L. (Bai Bian Dou)	Repair of intestinal barrier Inhibition of TLR5/MyD88/NF-κB pathway Regulation of inflammatory factors Antiapoptosis	Occludin, ZO-1↑, Claudin-1↓, MUC2↑ TLR5, MyD88, NF-κB↓ IL-1β, IL-6, TNF-α, IL-17A↓, IL-10↑, CRP↓ Bax↓, Bcl-2↑, Cleaved Caspase-3↓	Rao et al. (2022)
Sishen pills	*Psoralea corylifolia* L. (Bu Gu Zhi), *Myristica fragrans* Houtt. (Rou Dou Kou), *Schisandra chinensis* Turcz. (Wu Weu Zi), *Euodia rutaecarpa* Juss. (Wu Zhu Yu), *Ziziphus jujuba* Mill. (Da Zao) and *Zingiber officinale* Rosc. (Sheng Jiang)	Inhibition of miR-505-3p/E-cadherin pathway Regulation of DCs differentiation Regulation of inflammatory factors	miR-505-3p, CDH1↓ MHC-II, CD86, CD115, Flt3, SIRPα, F4/80, Ly6c, FcεRIα↓ IL-6, IL-1β, TNF-α↓	Huang et al. (2024b)
Shaoyao decoction	*Paeonia lactiflora* Pall. (Bai Shao), *Aucklandia lappa* Decne (Mu Xiang), *Cinnamomum cassia* (L.). J. Presl (Rou Gui), *Scutellaria baicalensis* Georgi (Huang Qin), *Areca catechu* L. (Bing Lang), *Rheum palmatum* L. (Da Huang), *Coptis chinensis* Franch. (Huang Lian), *Angelica sinensis* (Oliv.) Diels. (Dang Gui) and *Glycyrrhiza uralensis* Fisch. (Gan Cao)	Inhibition of IL-6/STAT3 pathway Restoration of Th17/Treg balance Regulation of inflammatory factors	IL-6, STAT3, p-STAT3, RORγt↓, Foxp3↑ CD4^+^IL-17^+^↓, CD4^+^Foxp3^+^↑ IL-6, IL-17↓, IL-10, TGF-β1↑	Wu et al. (2023a)
		Regulation of gut microbiota and its metabolites Activation of AHR/IL-22/STAT3 pathway Repair of intestinal barrier Regulation of inflammatory factors Antioxidant activity	*Akkermansia*, *Lactobacillus*↑, *Proteobacteria*↓, Trp, IA, IAA, IAld↑ AHR, CYP1A1, IL-22, p-STAT3/STAT3↑ Reg3β, Reg3γ↑, ZO-1、Occludin↑ IL-6, TNF-α, IL-1β↓, IL-10↑ MDA↓, T-AOC↑	Zhang et al. (2024d)
Sijunzi Decoction	*Panax ginseng* C. A. Mey. (Ren Shen), *Atractylodes macrocephala* Koidz. (Bai Zhu), *Wolfiporia cocos* (F.A. Wolf) Ryvarden and Gilb. (Fu Ling) and *Glycyrrhiza uralensis* Fisch. ex DC. (Gan Cao)	Regulation of gut microbiota and its metabolites Repair of intestinal barrier Regulation of inflammatory factors	*Proteobacteria*, *Escherichia-Shigella*↓, *Bacteroides*↑, TUDCA↑ ZO-1, Occludin, JAM-A↑, Claudin-1, Claudin-4, Claudin-11↑, Claudin-2↓, MUC2↑ TNF-α, IL-6, IL-1β, IFN-γ↓	Wu et al. (2023b)
		Regulation of gut microbiota Repair of intestinal barrier Regulation of inflammatory factors	*Akkermansia*, *Lachnospiraceae*↑, *Bacteroides*, *Helicobacter*↓ Occluding, ZO-1↑ IL-6, IL-1β, TNF-α↓	Li et al. (2024b)
Lizhong Decoction	*Zingiber officinale* Rosc. (Gan Jiang), *Panax ginseng* C. A. Mey. (Ren Shen), *Atractylodes macrocephala* Koidz (Bai Zhu) and *Glycyrrhiza uralensis* Fisch. (Gan Cao)	Inhibition of TLR4/NF-κB pathway Regulation of inflammatory factors Antioxidant activity Repair of intestinal barrier	TLR4, NF-κB↓ TNF-α, IFN-γ, IL-1β, IL-6↓, IL-4, IL-10↑ MPO↓, SOD↑ ZO-1, Occludin, Claudin-1↑	Shen et al. (2020)
		Regulation of gut microbiota Regulation of metabolism Regulation of inflammatory factors Antioxidant activity	*Clostridium sensu stricto 1*, *Enterobacter, Escherichia-Shigella*↓, *Blautia*, *Muribaculaceae_norank*, *Prevotellaceae UCG-001*, *Ruminiclostridium 9*↑ α-linolenic acid, stearidonic acid, L-tryptophan, cholic acid↑, adenosine, glycocholic acid, deoxycholic acid, lysoPC 18:0↓ TNF-α, IFN-γ, IL-1β, IL-6↓, IL-4, IL-10↑ MPO↓, SOD↑	Zou et al. (2020)
Huangqin Decoction	*Scutellaria baicalensis* Georgi (Huang Qin), *Paeonia lactiflora* Pall. (Shao Yao), *Glycyrrhiza uralensis* Fisch. (Gan Cao) and *Ziziphus jujuba* Mill. (Da Zao)	Regulation of gut microbiota Regulation of amino acid metabolites Activation of mTOR pathway Repair of intestinal barrier Antiapoptosis	*Firmicutes*↑, *Bacteroidetes*↓ L-Lysine, L-Leucine, L-Isoleucine, L-Glutamic acid↑; p-mTOR/mTOR, p-S6/S6, p-4E-BP1/4E-BP1↑ ZO-1, occluding, claudin-1, E-cadherin↑ Bcl-2↑, Bax, caspase-3↓	Li et al. (2022b)
Dahuang Mudan Decoction	*Rhei Radix et Rhizoma* (Da Huang), *Persicae Semen* (Tao Ren), *Natrii Sulfas* (Mang Xiao), *Moutan Cortex* (Mu Dan Pi) and *Semen Benincasae* (Dong Gua Zi)	Repair of intestinal barrier Regulation of ILC3 function Regulation of inflammatory factors	ZO-1, Occludin, Claudin-1↑ NCR^+^ILC3↑, NCR−ILC3↓ IL-22↑, IL-17A, GM-CSF↓	Huang et al. (2022)
Banxia Xiexin Decoction	*Pinellia ternata* (Thunb.) Breit. (Qing Ban Xia), *Scutellaria baicalensis* Georgi (Huang Qin), *Coptis chinensis* Franch. (Huang Lian), *Panax ginseng* C. A. Mey. (Ren Shen), *Zingiber officinale* Rosc. (Gan Jiang), *Glycyrrhiza uralensis* Fisch. (Chao Gan Cao) and *Ziziphus jujuba* Mill. (Da Zao)	Regulation of gut microbiota Regulation of metabolism Repair of intestinal barrier Regulation of inflammatory factors	*Desulfobacterota*, *Actinobacteriota*​, *g_norank_f_Muribaculaceae*, *Dubosiella*, *Akkermansia*, *Lactobacillus*​↑, *Faecalibaculum*, *Alloprevotella*, *Turicibacter*↓ Kynurenic acid, Uric acid, 13-HODE↓, 5-HT, Adenine, LysoPC, Indole↑ ZO-1, Occludin↑ MPO, TNF-α↓	Luo et al. (2024)
Baitouweng Decoction	*Radix pulsatilla* (Bai Tou Weng), *Cortex phellodendri* (Huang Bo), *Rhizoma coptidis* (Huang Lian) and *Cortex fraxini* (Qin Pi)	Restoration of Th17/Treg balance Regulation of inflammatory factors Repair of intestinal barrier Inhibition of ERK/NF-κB pathway Regulation of metabolism	CD4^+^ IL-17A^+^↓, CD4^+^CD25^+^Foxp3^+^↑ TNF-α, IL-1β, IL-6↓, IL-10↑ ZO-1, occludin↑; p-ERK, p-NF-κB↓, COX-2, iNOS↓ Acetate, Propionate, Isobutyric acid, Isovalerate↑	Miao et al. (2020)
		Inhibition of IL-6/STAT3 pathway Regulation of inflammatory factors Regulation of gut microbiota	IL-6, p-STAT3/STAT3↓ IL-6, IL-1β, TNF-α↓ *Firmicutes*/*Bacteroidetes*, *Proteobacteria*↓, *Escherichia-Shigella*↓, *Lactobacillus*, *Akkermansia*↑	Chen et al. (2021)

## 4 Conclusion and perspectives

In this paper, we systematically review the key mechanisms of immune dysregulation in the pathogenesis of UC, encompassing pathological aspects such as dysregulation of T-cell subsets, hypersecretion of pro-inflammatory cytokines, imbalance in the gut microbiota, and disruption of the intestinal barrier that synergistically contribute to the onset and progression of UC. Current studies have shown that immune dysregulation in UC is not confined solely to local inflammatory responses but also entails complex alterations in systemic immune regulation and metabolic networks. The overactivation of Th1 and Th17 cells and the insufficiency of Treg function amplify local pro-inflammatory signals; simultaneously, pro-inflammatory factors (e.g., TNF-α, IL-6, IL-17) activate signaling pathways such as NF-κB and MAPK, thereby aggravating epithelial cell apoptosis and TJ disruption, ultimately leading to a ‘leaky gut’ that facilitates the invasion of pathogenic bacteria and their products. In addition, gut microbiota imbalance not only alters bacterial composition but also modulates intestinal immune homeostasis via metabolites such as SCFAs, BAs, and Trp derivatives, whose dysregulation is closely associated with UC inflammation and prognosis. These findings offer novel insights into the pathomechanisms of UC and pave the way for precision treatment. As a traditional medical system, TCM has demonstrated unique advantages in the prevention and treatment of UC through multi-target and multi-pathway mechanisms, including immune regulation, anti-inflammatory repair, and flora remodeling. Both single compounds (e.g., berberine, matrine, curcumin) and compound formulations (e.g., Shenling Baizhu Powder, Sishen pills, Shaoyao decoction) ameliorate the inflammatory state and pathological alterations of UC by synergistically modulating the immune network, repairing intestinal barriers, and remodeling bacterial flora structure. This multilevel, multidimensional regulatory mechanism not only effectively alleviates local inflammation but also facilitates comprehensive disease intervention through systemic immune modulation.

Although clinical and experimental studies on TCM for UC treatment have yielded promising results, many urgent challenges remain. First, the mechanisms underlying TCM herbal formulas and their active ingredients are relatively complex, and the precise elucidation of their multi-target effects at the molecular level requires further investigation using systems biology, network pharmacology, and multi-omics technologies. Secondly, existing clinical trials are relatively small in scale, and there is a lack of large-scale, multicenter randomized controlled studies to validate the long-term efficacy and safety of TCM. In addition, the quality of Chinese herbal medicines varies across different origins and batches, necessitating the development of standardized formulations. Therefore, there is an urgent need to establish stringent quality control systems and standardized evaluation indices to enhance the reproducibility and reliability of Chinese medicine research. Modern molecular biology and systems biology can facilitate an in-depth analysis of the multi-component, multi-target regulation of the immune network by TCM, thereby revealing the scientific connotation. Simultaneously, integrating clinical data with multicenter randomized trials can further elucidate the synergistic effects of Chinese medicine when combined with conventional treatments, thereby providing a basis for developing individualized and precise treatment plans. Given the unique advantages of TCM in modulating intestinal flora, repairing intestinal barriers, and balancing immune cells, there is significant potential for exploring combination therapies. For example, TCM could be developed in conjunction with monoclonal antibodies targeting various cytokines or with inhibitors of STAT or JAK pathways. This approach could enhance the therapeutic efficacy of UC treatment by leveraging the distinct mechanisms of action of TCM alongside conventional therapies. In summary, the combined use of TCM with probiotics, metabolic modulators, and other novel therapeutic approaches warrants future exploration. Such multimodal interventions could advance UC treatment toward comprehensive regulation and improved patient outcomes.

In conclusion, this article has comprehensively explored the roles of dysregulation of T-cell subsets, hypersecretion of pro-inflammatory cytokines, imbalance in the gut microbiota, and disruption of the intestinal barrier in UC pathogenesis, thereby providing a solid theoretical foundation for elucidating its pathophysiological mechanisms. Moreover, Chinese medicine, with its unique multi-component, multi-target, and holistic regulatory advantages, has demonstrated irreplaceable efficacy in preventing and treating ulcerative colitis by modulating the immune network, repairing the intestinal barrier, and reshaping intestinal flora ecology. In the future, systems biology, multi-omics technologies, and large-scale clinical trials will further elucidate the multilevel regulatory mechanisms of TCM and promote the translation of ulcerative colitis treatment toward comprehensive intervention and individualized precision therapy.
